# Supplementary material for: Comparative diagnostic evaluation of real-time PCR and culture for detecting pathogens in podiatric wound infections
Source: Microbiol Spectr. 2025 Nov 21;14(1):e02649-25. doi: 10.1128/spectrum.02649-25 (PMC12772387; doi:10.1128/spectrum.02649-25)
Supplement: Data S6 — Python scripts and instructions. [file spectrum.02649-25-s0006.docx]

Python scripts and instructions

This document serves as a full reproducibility protocol for the organism-level probability and Latent Class Analysis models described in the manuscript. It consolidates all Python scripts, data inputs, execution instructions, and output validation checks. The analysis was performed on a MacBook Pro, and some example codes may need to be adjusted in the CMD terminal on Windows-based computers.

# A. organism-level probability model described in the manuscript

1- All required scripts and data should be placed in the same directory (e.g., ~/Downloads/WND_Prob/):

WND_Prob/
-Rebuilt_Modeling_Matrix_With_Expected_Gram_Morphology_editted.xlsx

- Final_Training_Organism_Level_Table_With_Gram_Scores.csv
- 062425_Updated_Master_Database_with_ABR_Genes. XLSX
- simulated_tn_predictions.csv
- infection_model_pipeline.py
- internal_model_validation.py
- morphology_model_validation.py
- morphology_model_validation_corrected.py
- apply_model_to_pcr_only.py
- infection_model_pipeline_scored.py
- plot_tn_simulation_histogram.py

outputs/ (generated automatically)

_ Master Data.XLSX should be renamed to 062425_Updated_Master_Database_with_ABR_Genes. XLSX

outputs/ (generated automatically)

# 1- Software Requirements

Python ≥ 3.10
Required libraries:

pip install pandas numpy scikit-learn matplotlib openpyxl

# 2. Step-by-Step Execution

| Step | Script | Outputs |
| --- | --- | --- |
| 1 | infection_model_pipeline.py | PCR_detected_predictions.csv |
| 2 | internal_model_validation.py | internal_validation_roc_curve.png, internal_validation_calibration_curve.png |
| 3 | morphology_model_validation.py | simulated_tn_predictions.csv, morphology_cutoffs.csv |
| 4 | morphology_model_validation_corrected.py | same as above |
| 5 | apply_model_to_pcr_only.py | PCR_only_predictions.csv |

# 3. Python Codes

- infection_model_pipeline.py

import pandas as pd

from sklearn.linear_model import LogisticRegression

from sklearn.pipeline import Pipeline

from sklearn.preprocessing import StandardScaler

from sklearn.calibration import CalibratedClassifierCV

# Step 1: Load matrix file

df = pd.read_excel("Rebuilt_Modeling_Matrix_With_Expected_Gram_Morphology_editted.xlsx")

# Step 2: Normalize columns

df['Expected_Gram_Morphology'] = df['Expected_Gram_Morphology'].str.upper()

for col in ['Ct', 'Ct_16S', 'GPC_score', 'GNR_score', 'GPR_score']:

df[col] = pd.to_numeric(df[col], errors='coerce')

# Step 3: Build TP + TN training set

df_tp = df[(df['Detected'] == 1) & (df['Is_True_Positive'] == 1)].copy()

df_tp['Training_Label'] = 1

df_tn = df[(df['Detected'] == 0) & (df['Is_True_Positive'] == 0)].copy()

df_tn['Training_Label'] = 0

df_train = pd.concat([df_tp, df_tn], ignore_index=True)

# Step 4: Mask Gram scores by morphology

df_train['GPC_model'] = df_train.apply(lambda r: r['GPC_score'] if r['Expected_Gram_Morphology'] == 'GPC' else 0, axis=1)

df_train['GNR_model'] = df_train.apply(lambda r: r['GNR_score'] if r['Expected_Gram_Morphology'] == 'GNR' else 0, axis=1)

# Step 5: Train calibrated logistic regression model

X_train = df_train[['Ct', 'Ct_16S', 'GPC_model', 'GNR_model']]

y_train = df_train['Training_Label']

base_model = Pipeline([

('scaler', StandardScaler()),

('logreg', LogisticRegression(solver='liblinear'))

])

model = CalibratedClassifierCV(base_model, method='sigmoid', cv=5)

model.fit(X_train, y_train)

# Step 6: Predict on all PCR-detected organisms

df_detected = df[df['Detected'] == 1].copy()

df_detected['GPC_model'] = df_detected.apply(lambda r: r['GPC_score'] if r['Expected_Gram_Morphology'] == 'GPC' else 0, axis=1)

df_detected['GNR_model'] = df_detected.apply(lambda r: r['GNR_score'] if r['Expected_Gram_Morphology'] == 'GNR' else 0, axis=1)

X_detected = df_detected[['Ct', 'Ct_16S', 'GPC_model', 'GNR_model']]

df_detected['Calibrated_Prob'] = model.predict_proba(X_detected)[:, 1]

# Step 7: Save results

df_detected.to_csv("PCR_detected_predictions.csv", index=False)

print("Done. Predictions saved to PCR_detected_predictions.csv")

- internal_model_validation.py

import pandas as pd

import matplotlib.pyplot as plt

from sklearn.pipeline import Pipeline

from sklearn.linear_model import LogisticRegression

from sklearn.preprocessing import StandardScaler

from sklearn.calibration import CalibratedClassifierCV, calibration_curve

from sklearn.metrics import confusion_matrix, roc_auc_score, roc_curve, classification_report, accuracy_score

# Load matrix file

df = pd.read_excel("Rebuilt_Modeling_Matrix_With_Expected_Gram_Morphology_editted.xlsx")

# Normalize and convert columns

df['Expected_Gram_Morphology'] = df['Expected_Gram_Morphology'].str.upper()

for col in ['Ct', 'Ct_16S', 'GPC_score', 'GNR_score', 'GPR_score']:

df[col] = pd.to_numeric(df[col], errors='coerce')

# Build training set: PCR + culture confirmed (TP) and PCR−/culture− (TN)

df_tp = df[(df['Detected'] == 1) & (df['Is_True_Positive'] == 1)].copy()

df_tp['Label'] = 1

df_tn = df[(df['Detected'] == 0) & (df['Is_True_Positive'] == 0)].copy()

df_tn['Label'] = 0

df_train = pd.concat([df_tp, df_tn], ignore_index=True)

# Apply morphology-aware masking

df_train['GPC_model'] = df_train.apply(lambda r: r['GPC_score'] if r['Expected_Gram_Morphology'] == 'GPC' else 0, axis=1)

df_train['GNR_model'] = df_train.apply(lambda r: r['GNR_score'] if r['Expected_Gram_Morphology'] == 'GNR' else 0, axis=1)

# Train model with Platt calibration

X = df_train[['Ct', 'Ct_16S', 'GPC_model', 'GNR_model']]

y = df_train['Label']

base_model = Pipeline([

('scaler', StandardScaler()),

('logreg', LogisticRegression(solver='liblinear'))

])

model = CalibratedClassifierCV(base_model, method='sigmoid', cv=5)

model.fit(X, y)

# Predict on training data

y_pred = model.predict(X)

y_prob = model.predict_proba(X)[:, 1]

# Confusion matrix and classification report

print("=== Confusion Matrix ===")

print(confusion_matrix(y, y_pred))

print("\n=== Classification Report ===")

print(classification_report(y, y_pred))

print(f"Accuracy: {accuracy_score(y, y_pred):.3f}")

print(f"AUC: {roc_auc_score(y, y_prob):.3f}")

# ROC Curve

fpr, tpr, _ = roc_curve(y, y_prob)

plt.figure()

plt.plot(fpr, tpr, label=f'AUC = {roc_auc_score(y, y_prob):.2f}')

plt.plot([0, 1], [0, 1], linestyle='--', color='gray')

plt.title('ROC Curve (Internal Validation)')

plt.xlabel('False Positive Rate')

plt.ylabel('True Positive Rate')

plt.legend()

plt.grid(True)

plt.tight_layout()

plt.savefig("internal_validation_roc_curve.png")

# Calibration Curve

plt.figure()

prob_true, prob_pred = calibration_curve(y, y_prob, n_bins=10, strategy='uniform')

plt.plot(prob_pred, prob_true, marker='o', label='Calibrated')

plt.plot([0, 1], [0, 1], linestyle='--', color='gray')

plt.title('Calibration Curve (Internal Validation)')

plt.xlabel('Predicted Probability')

plt.ylabel('Observed Frequency')

plt.legend()

plt.grid(True)

plt.tight_layout()

plt.savefig("internal_validation_calibration_curve.png")

print("Saved ROC and Calibration curves.")

- morphology_model_validation.py

import pandas as pd

from sklearn.calibration import CalibratedClassifierCV

from sklearn.linear_model import LogisticRegression

from sklearn.preprocessing import StandardScaler

from sklearn.pipeline import Pipeline

# Step 1: Load matrix and master files

df = pd.read_excel("Rebuilt_Modeling_Matrix_With_Expected_Gram_Morphology_editted.xlsx")

df_master = pd.read_excel("062425_Updated_Master_Database_with_ABR_Genes.xlsx")

# Normalize and convert columns

df['Expected_Gram_Morphology'] = df['Expected_Gram_Morphology'].str.upper()

for col in ['Ct', 'Ct_16S', 'GPC_score', 'GNR_score', 'GPR_score']:

df[col] = pd.to_numeric(df[col], errors='coerce')

# Build training set from confirmed positives and confirmed negatives

df_tp = df[(df['Detected'] == 1) & (df['Is_True_Positive'] == 1)].copy()

df_tp['Training_Label'] = 1

df_tn = df[(df['Detected'] == 0) & (df['Is_True_Positive'] == 0)].copy()

df_tn['Training_Label'] = 0

df_train = pd.concat([df_tp, df_tn], ignore_index=True)

# Morphology-based masking of features

df_train['GPC_model'] = df_train.apply(lambda r: r['GPC_score'] if r['Expected_Gram_Morphology'] == 'GPC' else 0, axis=1)

df_train['GNR_model'] = df_train.apply(lambda r: r['GNR_score'] if r['Expected_Gram_Morphology'] == 'GNR' else 0, axis=1)

# Fit a calibrated logistic regression model

X_train = df_train[['Ct', 'Ct_16S', 'GPC_model', 'GNR_model']]

y_train = df_train['Training_Label']

model = CalibratedClassifierCV(

Pipeline([

('scaler', StandardScaler()),

('logreg', LogisticRegression(solver='liblinear'))

]),

method='sigmoid',

cv=5

)

model.fit(X_train, y_train)

# Extract TN cases from master file with no culture-only organisms

tn_cases = df_master[

(df_master['Conventional_Classification'].str.upper() == 'TN') &

(df_master['Culture only organisms'].isna())

].copy()

# Convert morphology categories to scores

gram_map = {'Absent': 0, 'Low': 1, 'Medium': 2, 'High': 3}

tn_cases['GPC_score'] = tn_cases['Gram-positive cocci morphology'].map(gram_map)

tn_cases['GNR_score'] = tn_cases['Gram_Negative_Rods'].map(gram_map)

tn_cases['Ct_16S'] = pd.to_numeric(tn_cases['Ct_16S'], errors='coerce')

# Simulate high Ct PCR-only detections in TN cases

sim_data = []

for _, row in tn_cases.iterrows():

for morph in ['GPC', 'GNR']:

score = row[f'{morph}_score']

if pd.notna(score):

sim_data.append({

'Case_ID': row['Case_ID'],

'Morphology': morph,

'Ct': 35.0,

'Ct_16S': row['Ct_16S'],

'GPC_model': score if morph == 'GPC' else 0,

'GNR_model': score if morph == 'GNR' else 0

})

df_sim = pd.DataFrame(sim_data)

X_sim = df_sim[['Ct', 'Ct_16S', 'GPC_model', 'GNR_model']]

df_sim['Predicted_Prob'] = model.predict_proba(X_sim)[:, 1]

# Compute morphology-specific probability cutoffs

cutoffs = df_sim.groupby('Morphology')['Predicted_Prob'].max().reset_index()

cutoffs['Suggested_Cutoff'] = round(cutoffs['Predicted_Prob'] + 0.01, 3)

# Save outputs

df_sim.to_csv("simulated_tn_predictions.csv", index=False)

cutoffs.to_csv("morphology_cutoffs.csv", index=False)

print("Validation complete. Output saved as:")

print("- simulated_tn_predictions.csv")

print("- morphology_cutoffs.csv")

- morphology_model_validation_corrected.py

import pandas as pd

from sklearn.calibration import CalibratedClassifierCV

from sklearn.linear_model import LogisticRegression

from sklearn.preprocessing import StandardScaler

from sklearn.pipeline import Pipeline

# Load data

df = pd.read_excel("Rebuilt_Modeling_Matrix_With_Expected_Gram_Morphology_editted.xlsx")

df_master = pd.read_excel("062425_Updated_Master_Database_with_ABR_Genes.xlsx")

# Normalize primary matrix

df['Expected_Gram_Morphology'] = df['Expected_Gram_Morphology'].str.upper()

for col in ['Ct', 'Ct_16S', 'GPC_score', 'GNR_score', 'GPR_score']:

df[col] = pd.to_numeric(df[col], errors='coerce')

# Create training set: TPs and TNs

df_tp = df[(df['Detected'] == 1) & (df['Is_True_Positive'] == 1)].copy()

df_tp['Training_Label'] = 1

df_tn = df[(df['Detected'] == 0) & (df['Is_True_Positive'] == 0)].copy()

df_tn['Training_Label'] = 0

df_train = pd.concat([df_tp, df_tn], ignore_index=True)

# Morphology-specific score masking

df_train['GPC_model'] = df_train.apply(lambda r: r['GPC_score'] if r['Expected_Gram_Morphology'] == 'GPC' else 0, axis=1)

df_train['GNR_model'] = df_train.apply(lambda r: r['GNR_score'] if r['Expected_Gram_Morphology'] == 'GNR' else 0, axis=1)

# Train logistic regression with calibration

X_train = df_train[['Ct', 'Ct_16S', 'GPC_model', 'GNR_model']]

y_train = df_train['Training_Label']

model = CalibratedClassifierCV(

Pipeline([

('scaler', StandardScaler()),

('logreg', LogisticRegression(solver='liblinear'))

]),

method='sigmoid',

cv=5

)

model.fit(X_train, y_train)

# Fix: Correct gram columns for TN cases

gram_map = {'Absent': 0, 'Low': 1, 'Medium': 2, 'High': 3}

tn_cases = df_master[

(df_master['Conventional_Classification'].str.upper() == 'TN') &

(df_master['Culture only organisms'].isna())

].copy()

tn_cases['GPC_score'] = tn_cases['Gram_Positive_Cocci'].map(gram_map)

tn_cases['GNR_score'] = tn_cases['Gram_Negative_Rods'].map(gram_map)

tn_cases['Ct_16S'] = pd.to_numeric(tn_cases['Ct_16S'], errors='coerce')

# Simulate PCR-only detections in TNs

sim_data = []

for _, row in tn_cases.iterrows():

for morph in ['GPC', 'GNR']:

score = row[f'{morph}_score']

if pd.notna(score):

sim_data.append({

'Case_ID': row['Case_ID'],

'Morphology': morph,

'Ct': 35.0,

'Ct_16S': row['Ct_16S'],

'GPC_model': score if morph == 'GPC' else 0,

'GNR_model': score if morph == 'GNR' else 0

})

df_sim = pd.DataFrame(sim_data)

X_sim = df_sim[['Ct', 'Ct_16S', 'GPC_model', 'GNR_model']]

df_sim['Predicted_Prob'] = model.predict_proba(X_sim)[:, 1]

# Derive morphology-specific probability cutoffs

cutoffs = df_sim.groupby('Morphology')['Predicted_Prob'].max().reset_index()

cutoffs['Suggested_Cutoff'] = round(cutoffs['Predicted_Prob'] + 0.01, 3)

# Save results

df_sim.to_csv("simulated_tn_predictions.csv", index=False)

cutoffs.to_csv("morphology_cutoffs.csv", index=False)

print("Simulation complete. Output saved as:")

print("- simulated_tn_predictions.csv")

print("- morphology_cutoffs.csv")

- apply_model_to_pcr_only.py

import pandas as pd

from sklearn.pipeline import Pipeline

from sklearn.linear_model import LogisticRegression

from sklearn.preprocessing import StandardScaler

from sklearn.calibration import CalibratedClassifierCV

# Load data

df = pd.read_excel("Rebuilt_Modeling_Matrix_With_Expected_Gram_Morphology_editted.xlsx")

# Normalize columns

df['Expected_Gram_Morphology'] = df['Expected_Gram_Morphology'].str.upper()

for col in ['Ct', 'Ct_16S', 'GPC_score', 'GNR_score']:

df[col] = pd.to_numeric(df[col], errors='coerce')

# Training set: confirmed positives and confirmed negatives

df_tp = df[(df['Detected'] == 1) & (df['Is_True_Positive'] == 1)].copy()

df_tp['Label'] = 1

df_tn = df[(df['Detected'] == 0) & (df['Is_True_Positive'] == 0)].copy()

df_tn['Label'] = 0

df_train = pd.concat([df_tp, df_tn], ignore_index=True)

# Mask features by morphology

df_train['GPC_model'] = df_train.apply(lambda r: r['GPC_score'] if r['Expected_Gram_Morphology'] == 'GPC' else 0, axis=1)

df_train['GNR_model'] = df_train.apply(lambda r: r['GNR_score'] if r['Expected_Gram_Morphology'] == 'GNR' else 0, axis=1)

# Train calibrated model

X_train = df_train[['Ct', 'Ct_16S', 'GPC_model', 'GNR_model']]

y_train = df_train['Label']

model = CalibratedClassifierCV(

Pipeline([

('scaler', StandardScaler()),

('logreg', LogisticRegression(solver='liblinear'))

]),

method='sigmoid',

cv=5

)

model.fit(X_train, y_train)

# Apply to PCR-only (Detected == 1 but not culture confirmed)

df_pcr_only = df[(df['Detected'] == 1) & (df['Is_True_Positive'] == 0)].copy()

df_pcr_only['GPC_model'] = df_pcr_only.apply(lambda r: r['GPC_score'] if r['Expected_Gram_Morphology'] == 'GPC' else 0, axis=1)

df_pcr_only['GNR_model'] = df_pcr_only.apply(lambda r: r['GNR_score'] if r['Expected_Gram_Morphology'] == 'GNR' else 0, axis=1)

X_pcr = df_pcr_only[['Ct', 'Ct_16S', 'GPC_model', 'GNR_model']]

df_pcr_only['Predicted_Prob'] = model.predict_proba(X_pcr)[:, 1]

# Save output

df_pcr_only.to_csv("PCR_only_predictions.csv", index=False)

print("Saved predictions to PCR_only_predictions.csv")

# 4. Input Data Requirements

• Rebuilt_Modeling_Matrix_With_Expected_Gram_Morphology_editted.xlsx: Core organism-level dataset linking Ct values, Gram morphology, and truth labels (TP/TN).

• Final_Training_Organism_Level_Table_With_Gram_Scores.csv: Gram score calibration file used in infection_model_pipeline_scored.py.

• simulated_tn_predictions.csv: Intermediate file from morphology validation scripts (optional input).

• 062425_Updated_Master_Database_with_ABR_Genes.xlsx: Source of case-level metadata used for morphology correction (not required for primary training).

# 5. Data De-identification Statement

All datasets use Case_ID. The provided supplementary files are fully de-identified and safe for sharing.

# B. Latent Class Analysis

Note: All saved .py files should be saved in a folder along with input data file. Master data LCA.XLSX should be renamed to Master data.XLSX

cd ~/Desktop/LCA

python lca_broad_panel_pipeline.py \

  --xlsx "Master data.xlsx" \

  --sheet in \

  --outdir outputs \

  --bootstrap 300 \

  --seed 2025 \

  --multistart 100

Python script:

#!/usr/bin/env python3

"""

lca_broad_panel_pipeline.py

--------------------------------

End-to-end Python pipeline to:

1) Rebuild BROAD and PANEL binary test matrices from an Excel source (Master data.xlsx).

2) Fit 2-class latent class models (M1 = PCR+Culture; M3 = PCR+Culture+Gram) with multi-start EM.

3) Quantify uncertainty for M3 via parametric bootstrap with a locked seed for reproducibility.

4) Save all outputs (summaries, cross-tabs, bootstrap samples, CI tables).

Defaults mirror the Python runs you've seen in this session:

- BROAD:

PCR = 1 if ("Matched PCR and Culture organisms" OR "PCR-only (excluding flora & HTC)") has content

CULT = 1 if ("Matched" OR "Culture only organisms") has content

- PANEL (OFF-PANEL based: complement is ON-PANEL):

PCR = 1 if "Matched" has content OR PCR-only contains ANY ON-PANEL organism (i.e., NOT in OFF-PANEL list)

CULT = 1 if "Matched" has content OR Culture-only contains ANY ON-PANEL organism

Optionally, you can provide an explicit panel target list to define ON-PANEL detection by names

(case-insensitive exact match after trimming). When --panel-targets-file is supplied, PANEL is defined as:

PCR = 1 if PCR-only contains ANY name in that targets list

CULT = 1 if (Matched OR Culture-only) contains ANY name in that targets list

Usage (typical):

python lca_broad_panel_pipeline.py \

--xlsx Master data.xlsx --sheet in --outdir ./outputs \

--bootstrap 300 --seed 2025 --multistart 100

To use an explicit panel targets list (one target per line):

python lca_broad_panel_pipeline.py --panel-targets-file panel_targets.txt

Author: Your team

License: MIT

"""

from __future__ import annotations

import argparse

import json

from pathlib import Path

import sys

import time

import numpy as np

import pandas as pd

# -----------------------------

# Defaults / Constants

# -----------------------------

OFF_PANEL_DEFAULT = {

"pantoea agglomerans",

"group g streptococcus",

"pantoea species",

"prevotella bivia",

"peptostreptococcus species",

"prevotella denticola",

"pseudomonas fluorescens",

"bacteroides vulgatus",

"bacteroides fragilis group",

"corynebacterium striatum",

"yeast", # treat any "yeast..." as off-panel

}

# -----------------------------

# Utilities

# -----------------------------

def has_content(x) -> bool:

if pd.isna(x):

return False

s = str(x).strip().lower()

return s not in {"", "-", "–", "—", "na", "n/a", "none", "null"}

def parse_orgs(cell) -> list[str]:

if pd.isna(cell):

return []

out = []

txt = str(cell).replace("\n", ";")

for raw in txt.split(";"):

for piece in str(raw).split(","):

name = piece.strip().lower()

if name and name not in {"-", "–", "—", "na", "n/a", "none", "null"}:

out.append(name)

return out

def any_on_panel_by_offlist(orgs: list[str], off_panel: set[str]) -> bool:

for o in orgs:

base = "yeast" if o.startswith("yeast") else o

if base not in off_panel:

return True

return False

def any_in_targets(orgs: list[str], targets: set[str]) -> bool:

for o in orgs:

# exact name match (case-insensitive) after trimming

if o in targets:

return True

return False

# -----------------------------

# Input construction

# -----------------------------

def build_included_from_excel(

xlsx_path: Path,

sheet: str = "in",

panel_targets: set[str] | None = None,

off_panel: set[str] | None = None,

) -> pd.DataFrame:

"""

Returns DataFrame with columns:

Case_No, PCR_BROAD, CULT_BROAD, GRAM, PCR_PANEL, CULT_PANEL

"""

off_panel = off_panel or OFF_PANEL_DEFAULT

df = pd.read_excel(xlsx_path, sheet_name=sheet).copy()

# Prepare source columns

pcr_match_col = "Matched PCR and Culture organisms"

pcr_only_col = "PCR-only (excluding flora & HTC)"

cult_only_col = "Culture only organisms"

# Gram flag: any of the three morphology fields non-blank

gram_cols = ["Gram_Positive_Cocci", "Gram_Positive_Rods", "Gram_Negative_Rods"]

df["GRAM"] = df[gram_cols].apply(lambda r: int(any(has_content(v) for v in r)), axis=1)

# BROAD definitions (Python-session defaults)

df["PCR_BROAD"] = (df[pcr_match_col].apply(has_content) | df[pcr_only_col].apply(has_content)).astype(int)

df["CULT_BROAD"] = (df[pcr_match_col].apply(has_content) | df[cult_only_col].apply(has_content)).astype(int)

# PANEL definitions

match_has = df[pcr_match_col].apply(has_content)

pcr_only_lists = df[pcr_only_col].apply(parse_orgs)

cult_only_lists = df[cult_only_col].apply(parse_orgs)

if panel_targets is None:

# OFF-PANEL complement logic (default in this session)

pcr_onpanel = match_has | pcr_only_lists.apply(lambda L: any_on_panel_by_offlist(L, off_panel))

cult_onpanel = match_has | cult_only_lists.apply(lambda L: any_on_panel_by_offlist(L, off_panel))

else:

# Explicit targets: PCR uses PCR-only; CULT uses Matched + Culture-only combined

# (closer to your R prototype)

pcr_onpanel = pcr_only_lists.apply(lambda L: any_in_targets(L, panel_targets))

combined_lists = (df[pcr_match_col].fillna("").astype(str) + " ; " +

df[cult_only_col].fillna("").astype(str)).apply(parse_orgs)

cult_onpanel = combined_lists.apply(lambda L: any_in_targets(L, panel_targets))

df["PCR_PANEL"] = pcr_onpanel.astype(int)

df["CULT_PANEL"] = cult_onpanel.astype(int)

included_mask = df["Inclusion_Status"].astype(str).str.strip().str.lower() == "included"

inc = df.loc[included_mask, ["Case_No", "PCR_BROAD", "CULT_BROAD", "GRAM", "PCR_PANEL", "CULT_PANEL"]].copy()

return inc

# -----------------------------

# LCA EM (2-class, binary) + helpers

# -----------------------------

def fit_lca_em_binary(Y: np.ndarray, n_classes: int = 2, max_iter: int = 2000, tol: float = 1e-7,

seed: int | None = None, init: dict | None = None, eps: float = 1e-10) -> dict:

if seed is not None:

np.random.seed(seed)

N, J = Y.shape

K = n_classes

if init is None:

pi = np.random.dirichlet(np.ones(K))

theta1 = np.random.uniform(0.15, 0.95, size=(K, J))

theta = np.zeros((K, J, 2))

theta[:, :, 1] = theta1

theta[:, :, 0] = 1 - theta1

else:

pi = init["pi"].copy()

theta = init["theta"].copy()

loglik_old = -np.inf

for it in range(max_iter):

# E-step: responsibilities

log_gamma = np.zeros((N, K))

for k in range(K):

log_p = np.zeros(N)

for j in range(J):

p1 = np.clip(theta[k, j, 1], eps, 1 - eps)

p0 = np.clip(theta[k, j, 0], eps, 1 - eps)

log_p += Y[:, j] * np.log(p1) + (1 - Y[:, j]) * np.log(p0)

log_gamma[:, k] = np.log(np.clip(pi[k], eps, 1.0)) + log_p

# normalize

m = log_gamma.max(axis=1, keepdims=True)

gamma = np.exp(log_gamma - m)

gamma = gamma / gamma.sum(axis=1, keepdims=True)

# M-step

Nk = gamma.sum(axis=0) + eps

pi = Nk / (N + K * eps)

for k in range(K):

for j in range(J):

num1 = np.sum(gamma[:, k] * Y[:, j]) + eps

denom = Nk[k] + 2 * eps

theta[k, j, 1] = num1 / denom

theta[k, j, 0] = 1 - theta[k, j, 1]

# Log-likelihood

loglik = 0.0

for n in range(N):

s = 0.0

for k in range(K):

p = pi[k]

for j in range(J):

p *= theta[k, j, Y[n, j]]

s += p

loglik += np.log(np.clip(s, eps, 1.0))

if abs(loglik - loglik_old) < tol:

break

loglik_old = loglik

return {"pi": pi, "theta": theta, "loglik": float(loglik), "gamma": gamma, "iters": it + 1}

def reorder_classes(model: dict) -> dict:

"""Make class 1 the 'disease' class (higher mean positivity across tests)."""

pi, theta = model["pi"], model["theta"]

mean_pos = theta[:, :, 1].mean(axis=1)

if mean_pos[1] >= mean_pos[0]:

return model

pi_swapped = np.array([pi[1], pi[0]])

theta_swapped = np.stack([theta[1], theta[0]], axis=0)

gamma_swapped = model["gamma"][:, [1, 0]]

return {"pi": pi_swapped, "theta": theta_swapped, "loglik": model["loglik"], "gamma": gamma_swapped, "iters": model["iters"]}

def multi_start(Y: np.ndarray, n_starts: int = 100, seeds: list[int] | None = None) -> tuple[dict, list[tuple[int, float]]]:

best = None

logs: list[tuple[int, float]] = []

for s in range(n_starts):

seed = None if seeds is None else seeds[s % len(seeds)]

m = fit_lca_em_binary(Y, seed=seed, tol=1e-7, max_iter=3000)

m = reorder_classes(m)

logs.append((s, m["loglik"]))

if (best is None) or (m["loglik"] > best["loglik"]):

best = m

return best, logs

def summarize_model(model: dict, J: int, test_names: list[str]) -> dict:

pi = model["pi"]; theta = model["theta"]; loglik = model["loglik"]

N = model["gamma"].shape[0]

p = (2 - 1) + 2 * J

AIC = 2 * p - 2 * loglik

BIC = p * np.log(N) - 2 * loglik

res = {

"N": int(N), "LogLik": float(loglik), "AIC": float(AIC), "BIC": float(BIC),

"pi_class0": float(pi[0]), "pi_class1": float(pi[1]),

}

for j, name in enumerate(test_names):

res[f"{name}_sens"] = float(theta[1, j, 1])

res[f"{name}_spec"] = float(theta[0, j, 0])

return res

def cross_tab(Y: np.ndarray) -> pd.DataFrame:

df = pd.DataFrame(Y[:, :2], columns=["PCR", "CULT"])

ct = df.value_counts().sort_index()

rows = []

for a in (0, 1):

for b in (0, 1):

rows.append({"PCR": a, "CULT": b, "Count": int(ct.get((a, b), 0))})

return pd.DataFrame(rows)

def simulate_from(pi: np.ndarray, theta: np.ndarray, N: int, rng: np.random.RandomState) -> np.ndarray:

z = rng.choice([0, 1], size=N, p=pi)

J = theta.shape[1]

Y = np.zeros((N, J), dtype=int)

for j in range(J):

p1 = theta[z, j, 1]

Y[:, j] = rng.binomial(1, p1)

return Y

def bootstrap_parametric(fit_model: dict, R: int = 300, seed: int = 2025) -> tuple[pd.DataFrame, pd.DataFrame, float]:

# Lock seed for identical intervals per run

np.random.seed(seed)

pi_hat = fit_model["pi"]

th_hat = fit_model["theta"]

N = fit_model["gamma"].shape[0]

J = th_hat.shape[1]

metrics = []

t0 = time.time()

for r in range(R):

rng = np.random.RandomState(np.random.randint(0, 2**31 - 1))

Yb = simulate_from(pi_hat, th_hat, N, rng)

# jittered init near MLE for stability

jitter = 0.02

pi0 = np.clip(pi_hat + rng.normal(0, jitter, size=pi_hat.shape), 1e-6, 1.0); pi0 = pi0 / pi0.sum()

th0 = th_hat.copy()

for k in range(2):

for j in range(J):

t = np.clip(th0[k, j, 1] + rng.normal(0, jitter), 0.02, 0.98)

th0[k, j, 1] = t; th0[k, j, 0] = 1 - t

m = fit_lca_em_binary(Yb, init={"pi": pi0, "theta": th0}, tol=1e-7, max_iter=1500)

m = reorder_classes(m)

row = {"pi_class0": m["pi"][0], "pi_class1": m["pi"][1]}

names = ["PCR", "CULT", "GRAM"][:J]

for j, name in enumerate(names):

row[f"{name}_sens"] = m["theta"][1, j, 1]

row[f"{name}_spec"] = m["theta"][0, j, 0]

metrics.append(row)

boot = pd.DataFrame(metrics)

def ci(s: pd.Series) -> pd.Series:

return pd.Series({

"lower": np.percentile(s, 2.5),

"upper": np.percentile(s, 97.5),

"mean": s.mean(),

"sd": s.std(ddof=1),

})

cis = boot.apply(ci).T

elapsed = time.time() - t0

return boot, cis, elapsed

# -----------------------------

# Main driver

# -----------------------------

def main():

ap = argparse.ArgumentParser(description="LCA pipeline for BROAD and PANEL scenarios (Python only).")

ap.add_argument("--xlsx", type=str, default="LCA.xlsx", help="Path to Excel source (default: LCA.xlsx).")

ap.add_argument("--sheet", type=str, default="in", help="Sheet name (default: in).")

ap.add_argument("--outdir", type=str, default=".", help="Output directory (default: current dir).")

ap.add_argument("--bootstrap", type=int, default=300, help="Bootstrap replicates for M3 (default: 300).")

ap.add_argument("--seed", type=int, default=2025, help="Seed for parametric bootstrap (default: 2025).")

ap.add_argument("--multistart", type=int, default=100, help="Multi-start runs per model (default: 100).")

ap.add_argument("--panel-targets-file", type=str, default=None,

help="Optional text file with explicit panel targets (one per line). "

"If supplied, PANEL uses this list instead of OFF-PANEL complement.")

ap.add_argument("--off-panel-file", type=str, default=None,

help="Optional text file with OFF-PANEL organisms (one per line). Overrides built-in list.")

args = ap.parse_args()

xlsx_path = Path(args.xlsx)

outdir = Path(args.outdir)

outdir.mkdir(parents=True, exist_ok=True)

# Load optional lists

panel_targets = None

if args.panel_targets_file:

targets = Path(args.panel_targets_file).read_text(encoding="utf-8").splitlines()

targets = [t.strip().lower() for t in targets if t.strip()]

panel_targets = set(targets)

off_panel = OFF_PANEL_DEFAULT

if args.off_panel_file:

op = Path(args.off_panel_file).read_text(encoding="utf-8").splitlines()

op = [t.strip().lower() for t in op if t.strip()]

off_panel = set(op)

# Build included data

inc = build_included_from_excel(xlsx_path, sheet=args.sheet, panel_targets=panel_targets, off_panel=off_panel)

# Save scenario matrices

broad = inc[["Case_No", "PCR_BROAD", "CULT_BROAD", "GRAM"]].rename(columns={"PCR_BROAD": "PCR", "CULT_BROAD": "CULT"})

panel = inc[["Case_No", "PCR_PANEL", "CULT_PANEL", "GRAM"]].rename(columns={"PCR_PANEL": "PCR", "CULT_PANEL": "CULT"})

broad.to_csv(outdir / "LCA_inputs_included_BROAD.csv", index=False)

panel.to_csv(outdir / "LCA_inputs_included_PANEL.csv", index=False)

master_rows = []

for label, df in [("BROAD", broad), ("PANEL", panel)]:

# ----- M1 -----

Y1 = df[["PCR", "CULT"]].astype(int).values

best1, logs1 = multi_start(Y1, n_starts=args.multistart, seeds=list(range(args.seed, args.seed + args.multistart)))

sum1 = summarize_model(best1, J=2, test_names=["PCR", "CULT"])

xt1 = cross_tab(Y1)

pd.DataFrame([sum1]).to_csv(outdir / f"M1_{label}_summary.csv", index=False)

xt1.to_csv(outdir / f"M1_{label}_xtab.csv", index=False)

pd.DataFrame(logs1, columns=["start_idx", "loglik"]).sort_values("loglik", ascending=False).to_csv(outdir / f"M1_{label}_runlogs.csv", index=False)

# ----- M3 -----

Y3 = df[["PCR", "CULT", "GRAM"]].astype(int).values

best3, logs3 = multi_start(Y3, n_starts=args.multistart, seeds=list(range(args.seed, args.seed + args.multistart)))

sum3 = summarize_model(best3, J=3, test_names=["PCR", "CULT", "GRAM"])

pd.DataFrame([sum3]).to_csv(outdir / f"M3_{label}_summary.csv", index=False)

pd.DataFrame(logs3, columns=["start_idx", "loglik"]).sort_values("loglik", ascending=False).to_csv(outdir / f"M3_{label}_runlogs.csv", index=False)

# ----- Bootstrap (M3) -----

boot3, cis3, elapsed = bootstrap_parametric(best3, R=args.bootstrap, seed=args.seed)

boot3.to_csv(outdir / f"M3_{label}_bootstrap_samples.csv", index=False)

# Combine point + CI for convenience

point_series = pd.Series({

"pi_class0": sum3["pi_class0"], "pi_class1": sum3["pi_class1"],

"PCR_sens": sum3["PCR_sens"], "PCR_spec": sum3["PCR_spec"],

"CULT_sens": sum3["CULT_sens"], "CULT_spec": sum3["CULT_spec"],

"GRAM_sens": sum3["GRAM_sens"], "GRAM_spec": sum3["GRAM_spec"],

}, name="point")

quick = pd.concat([point_series, cis3], axis=1)

quick.to_csv(outdir / f"M3_{label}_summary_with_CIs.csv")

# Save CI matrix in multiple formats

cis3.to_pickle(outdir / f"M3_{label}_bootstrap_CIs.pkl")

cis3.to_json(outdir / f"M3_{label}_bootstrap_CIs.json", orient="table")

# Add to master

master_rows += [

{"Scenario": label, "Model": "M1", **sum1},

{"Scenario": label, "Model": "M3", **sum3},

]

# Master summary

pd.DataFrame(master_rows).to_csv(outdir / "LCA_summary_all.csv", index=False)

# Save a small run manifest

manifest = {

"xlsx": str(xlsx_path),

"sheet": args.sheet,

"outdir": str(outdir.resolve()),

"bootstrap": args.bootstrap,

"seed": args.seed,

"multistart": args.multistart,

"panel_mode": "explicit_targets" if panel_targets is not None else "off_panel_complement",

"panel_targets_file": args.panel_targets_file,

"off_panel_file": args.off_panel_file,

"timestamp": time.strftime("%Y-%m-%d %H:%M:%S"),

}

Path(outdir / "RUN_MANIFEST.json").write_text(json.dumps(manifest, indent=2), encoding="utf-8")

print("Pipeline finished. Outputs written to:", str(outdir.resolve()))

if __name__ == "__main__":

try:

main()

except Exception as e:

print("ERROR:", e, file=sys.stderr)

sys.exit(1)
