# Supplementary material for: Comparative diagnostic evaluation of real-time PCR and culture for detecting pathogens in podiatric wound infections
Source: Microbiol Spectr. 2025 Nov 21;14(1):e02649-25. doi: 10.1128/spectrum.02649-25 (PMC12772387; doi:10.1128/spectrum.02649-25)
Supplement: Supplemental figures — Fig. S1 to S3. [file spectrum.02649-25-s0007.pdf]

Supplementary Figures:

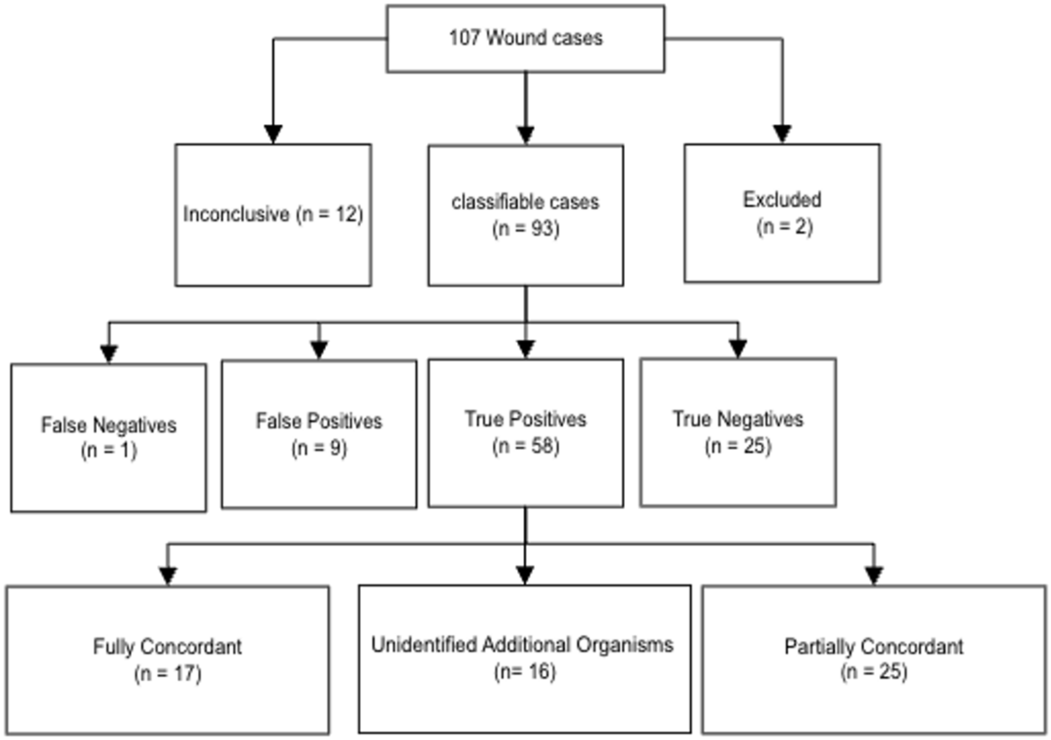

Figure S1. Flowchart illustrating the classification of 107 wound infection cases based on conventional diagnostic criteria. Of the total 107 cases, two were excluded due to mixed or unidentified results, and 12 were deemed inconclusive due to limitations in aerobic or anaerobic culture. The remaining 93 cases were classified into True Positives (n=58), False Positives (n = 9), False Negatives (n=1), and True Negatives (n = 29), False Negatives (n=1), and True Negatives (Fully Concordant (n=17), Partially Concordant (n=25), and cases with Unidentified Additional Organisms (n=16).

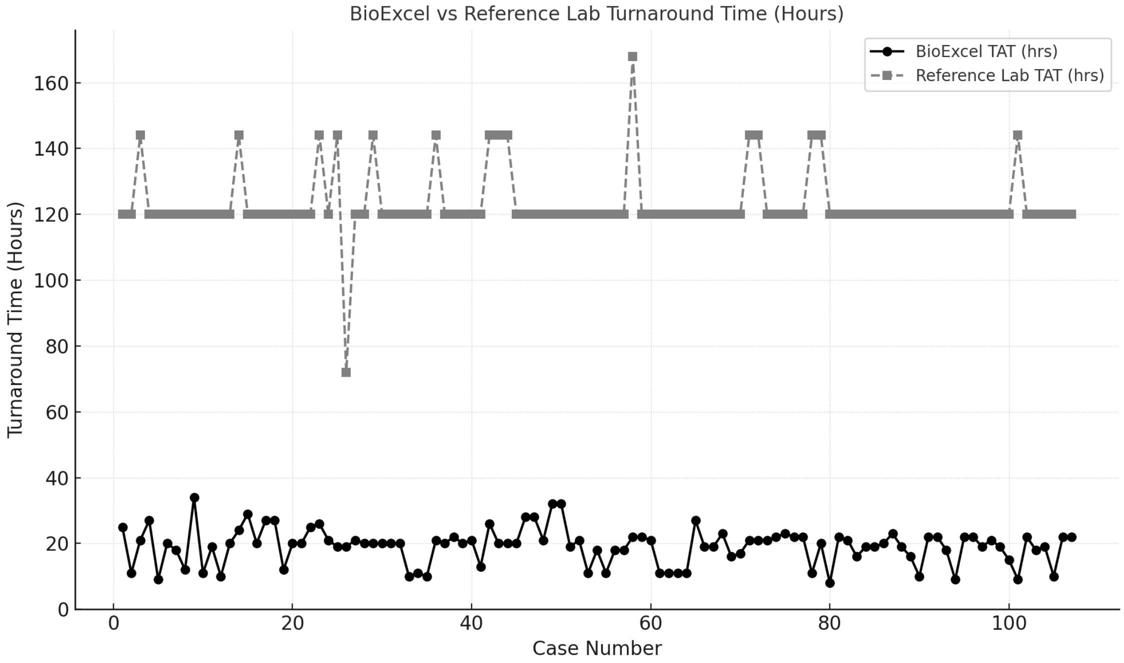

Figure S2. Turnaround time (TAT) comparison between PCR and culture. Case-wise turnaround times (TATs) for PCR (median 20 h; interquartile range [IQR], 4.5 h) and the reference laboratory's culture workflow (median 120 h; IQR, 0 h). Wilcoxon signed-rank test, $p = 2.57 \times 10^{-19}$. The reference lab's fixed mid-distribution TAT (IQR = 0) suggests batch or scheduled reporting; PCR shows modest variability around a ~20-hour median.

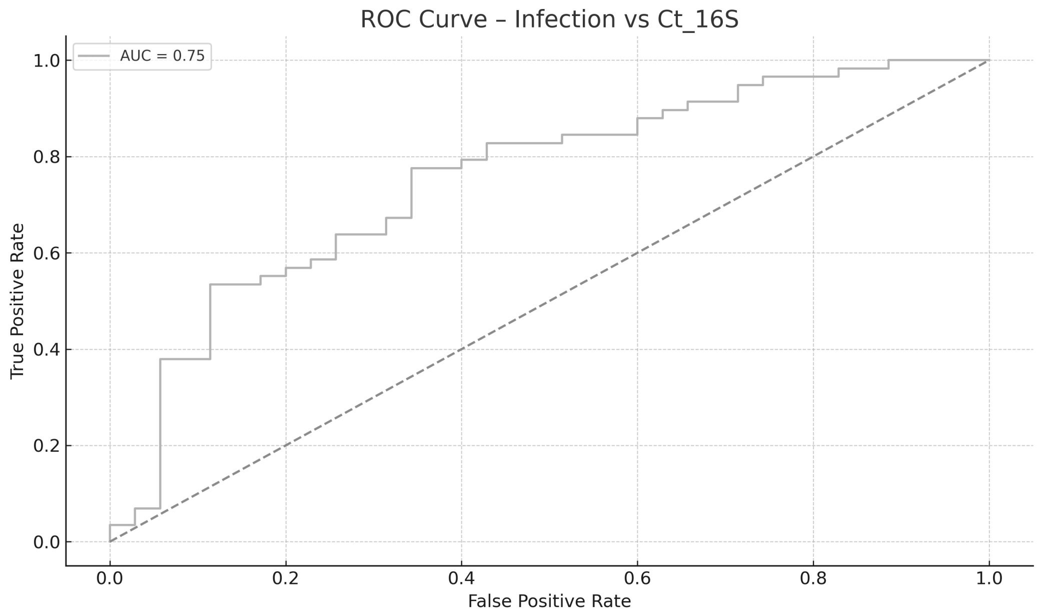

Figure S3. Receiver operating characteristic (ROC) curve showing the predictive value of pan-bacterial burden ($Ct_{16}S$) for infection status. AUC = 0.75, with Youden's optimal threshold at Ct ≤ 18.4 (sensitivity = 78%, specificity = 66%).
