# Supplementary material for: Comparative diagnostic evaluation of real-time PCR and culture for detecting pathogens in podiatric wound infections
Source: Microbiol Spectr. 2025 Nov 21;14(1):e02649-25. doi: 10.1128/spectrum.02649-25 (PMC12772387; doi:10.1128/spectrum.02649-25)
Supplement: Supplemental tables — Tables S1 to S13. [file spectrum.02649-25-s0008.pdf]

Supplementary Table S1. Distribution of Sample Types in the Wound Infection Cohort

This table summarizes the anatomical and procedural types of samples collected from 107 wound infection cases analyzed by both PCR and culture. The majority were superficial or unspecified swabs, followed by ulcer-targeted swabs and tissue biopsies.

Sample Type	Count	Percentage (%)
Swab (unspecified)	49	45.8
Swab (ulcer)	37	34.6
Biopsy, Shave	6	5.6
Swab (abscess)	5	4.7
Bone	2	1.9
Swab, deep to bone	2	1.9
Excision (e.g, lesion, skin, soft tissues)	1	0.9
Swab (ulcer), Deep to subcutaneous tissue	1	0.9
Swab (ulcer), Deep to muscle	1	0.9
Aspirate	1	0.9
Biopsy, Shave (soft tissue)	1	0.9
Swab (Blister)	1	0.9

Supplementary Table S2. Comparison of Turnaround Time Between Bioexcel and Reference Laboratory

This table summarizes the turnaround time (TAT) in hours for PCR (BioExcel Diagnostics) and culture (the reference laboratory) across 107 wound infection cases. Bioexcel showed notably faster reporting, with a median TAT of 20 hours compared to 120 hours for the reference lab. Statistical analysis confirmed this difference as highly significant. The IQR (Interquartile Range) indicates the spread of the middle 50% of the dataset. The interquartile range (IQR) of turnaround time (TAT) was 4.5 hours for Bioexcel and 0 hours for the reference laboratory.

This indicates that PCR TAT varied slightly around the median (20 hours), reflecting flexible and responsive processing within a constrained window.

In contrast, the reference laboratory had a fixed TAT of 120 hours across most cases, as evidenced by an IQR of 0, suggesting batch-based or scheduled reporting, with no variability across the middle 50% of samples.

This stark contrast underscores the PCR workflow's operational agility and highlights potential delays inherent to centralized or periodic reporting workflows used by the reference lab for culture.

- Test used: Wilcoxon signed-rank test (non-parametric, paired)
- p-value: 2.57×10^{-19}

Statistic	PCR TAT (Hours)	Culture TAT (Hours)
count	107.0	107.0
mean	19.21	123.14
std	5.47	10.47
min	8.0	72.0
25%	17.5	120.0
50%	20.0	120.0
75%	22.0	120.0
max	34.0	168.0
IQR	4.5	0.0

Table S3. PCR Detection Rate by Organism in all specimens (n= 103), including organisms excluded from the analyses, i.e Flora and Hard-to-Culture (HTC) organisms

Organism	Detection Count (n)	Detection Rate (%)	Flora/HTC
Coagulase-negative Staphylococci species	60	64.52	Yes
Staphylococcus aureus	38	40.86	No
Finegoldia magna	31	33.33	Yes
Enterococcus faecalis	30	32.26	No
Peptoniphilus Species	22	23.66	Yes
Pseudomonas aeruginosa	14	15.05	No
Staphylococcus lugdunensis	11	11.83	No
*Streptococcus agalactiae**	10	10.75	No
Actinotignum schaalii	8	8.60	Yes
Escherichia coli	7	7.53	No
Klebsiella pneumoniae	6	6.45	No
Enterobacter cloacae	6	6.45	No
Klebsiella oxytoca	5	5.38	No
Proteus mirabilis	5	5.38	No
Viridans streptococci	5	5.38	No
Peptostreptococcus anaerobius	4	4.30	Yes
Stenotrophomonas maltophilia	4	4.30	No
Morganella morganii	4	4.30	No
Enterococcus faecium	3	3.23	No
Bacteroides fragilis	3	3.23	No
Citrobacter koseri	2	2.15	No
Serratia marcescens	2	2.15	No
Aerococcus urinae	2	2.15	No
Acinetobacter baumannii	2	2.15	No
Candida glabrata	2	2.15	No

Candida albicans	2	2.15	No
C. novyi A/B_C. septicum_H. histolytica_P. sordellii group	1	1.08	No
Citrobacter freundii	1	1.08	No
Candida parapsilosis	1	1.08	No
Clostridium perfringens	1	1.08	No
Proteus vulgaris	1	1.08	No
Corynebacterium riegelii	1	1.08	No

* The manuscript's mention of 11 *Streptococcus agalactiae* detections is accurate across the full dataset, but only 10 are part of the 93 included cases analyzed for performance metrics.

Table S4. Culture Detection Rate by Organism

Organism	Detection Count (n)	Detection Rate (%)
Staphylococcus aureus	36	38.71
Pseudomonas aeruginosa	15	16.13
Streptococcus agalactiae	5	5.38
Escherichia coli	4	4.30
Yeast	4	4.30
Bacteroides fragilis	3	3.23
Group g Streptococcus*	2	2.15
Prevotella bivia*	1	1.08
Finegoldia magna	1	1.08
Morganella morganii	1	1.08
Klebsiella pneumoniae	1	1.08
Serratia marcescens	1	1.08
Prevotella denticola*	1	1.08
Pantoea agglomerans*	1	1.08
Corynebacterium striatum*	1	1.08
Bacteroides fragilis group*	1	1.08
Bacteroides vulgatus*	1	1.08
pseudomonas fluorescens*	1	1.08
Staphylococcus lugdunensis	1	1.08

* Organisms not covered by the PCR panel

Supplemntary Table S5. Cases with PCR only Organisms							
Case_No	Matched PCR and Culture organisms	PCR-only (excluding flora & HTC)	PCR-only organism	Conventional Classification	Concordance	Ct_16S_Category	ABR Gene
1	Escherichia coli	*enterobacter cloacae; Enterococcus faecium*	2	TP	Partially Concordant	Extra High	Erm(A)/Erm(B); Tet(M); Van Group; ampC
7		*Enterococcus faecalis*	1	FP	Discordant	Extra High	Erm(A)/Erm(B); Tet(M); mecA
8	Candida parapsilosis	*candida albicans; Enterococcus faecalis*	2	TP	Partially Concordant	Medium to High	Erm(A)/Erm(B); Mef(A); Tet(M); mecA
12	Staphylococcus aureus	*Enterococcus faecalis; Streptococcus agalactiae*	2	TP	Partially Concordant	Extra High	Erm(A)/Erm(B); Tet(M); mecA
13		*Enterococcus faecalis; proteus mirabilis*	2	FP	Discordant	Extra High	Erm(A)/Erm(B); Tet(M)
15	Staphylococcus aureus	*Enterococcus faecalis; proteus mirabilis*	2	TP	Partially Concordant	Extra High	Erm(A)/Erm(B); Tet(M); mecA
18	Pseudomonas aeruginosa	*Enterococcus faecalis*	1	TP	Partially Concordant	Extra High	Tet(M); mecA
19	Staphylococcus aureus; Streptococcus agalactiae	*Enterococcus faecalis; morganella morganii; proteus mirabilis; proteus vulgaris*	4	TP	Partially Concordant	Extra High	Erm(A)/Erm(B); Mef(A); Tet(M); mecA
22	Staphylococcus aureus	*Enterococcus faecalis*	1	TP	Partially Concordant	Extra High	Erm(A)/Erm(B); Tet(M); mecA
25	Staphylococcus aureus; Streptococcus agalactiae	*enterobacter cloacae; Enterococcus faecalis; viridans streptococci*	3	TP	UAO	Extra High	AAC(6)-Ib (aac(6)-Ib),Ant(3),APH(3)-VIb (aph(3)-VI; Erm(A)/Erm(B); Mef(A); Sul Group; Tet(M); ampC; mecA
28	Staphylococcus aureus; Bacteroides fragilis	*Enterococcus faecalis*	1	TP	Partially Concordant	Extra High	Sul Group; Tet(M); mecA
29		*citrobacter koseri; Enterococcus faecalis; Klebsiella oxytoca; Staphylococcus aureus*	4	FP	Discordant	Extra High	Erm(A)/Erm(B); Mef(A); Sul Group; Tet(A)/Tet(B)/Tet(S); Tet(M); Van Group; mecA
30		*Staphylococcus lugdunensis*	1	FP	Discordant	Extra High	Tet(M); mecA
33		*Staphylococcus lugdunensis*	1	FP	Discordant	Extra High	mecA
36	Pseudomonas aeruginosa; Staphylococcus aureus	*Peptostreptococcus anaerobius; Staphylococcus lugdunensis; Streptococcus agalactiae; viridans streptococci*	4	TP	Partially Concordant	Extra High	Erm(A)/Erm(B); Mef(A); Tet(M); mecA
41	Staphylococcus aureus	*Enterococcus faecalis; Klebsiella pneumoniae; viridans streptococci*	3	TP	Partially Concordant	Extra High	Erm(A)/Erm(B); Mef(A); SHV; Tet(M); mecA
44		*aerococcus urinae*	1	FP	Discordant	Extra High	Tet(M); mecA
45	Staphylococcus aureus	*Klebsiella pneumoniae*	1	TP	UAO	Extra High	SHV; mecA
46	Pseudomonas aeruginosa; Bacteroides fragilis	*citrobacter koseri; morganella morganii; serratia marcescens*	3	TP	UAO	Extra High	DHA; Erm(A)/Erm(B); Mef(A); Tet(M)
47	Pseudomonas aeruginosa	*Enterococcus faecalis; proteus mirabilis*	2	TP	Partially Concordant	Extra High	Erm(A)/Erm(B); Tet(M); mecA

Supplemntary Table S5. Cases with PCR only Organisms-continued

Case_No	Matched PCR and Culture organisms	PCR-only (excluding flora & HTC)	PCR-only organism	Conventional Classification	Concordance	Ct_16S_Category	ABR Gene
48	Staphylococcus aureus	*Enterococcus faecalis; stenotrophomonas maltophilia; viridans streptococci*	5	TP	Partially Concordant	Extra High	
49	Escherichia coli	*Staphylococcus lugdunensis*	1	TP	Partially Concordant	Extra High	mecA
52	Pseudomonas aeruginosa	*enterobacter cloacae; Enterococcus faecalis; Klebsiella pneumoniae*	3	TP	UAO	Extra High	SHV; Tet(M); ampC
54		*enterobacter cloacae*	1	FP	Discordant	Extra High	ampC; mecA
55	Staphylococcus aureus	*clostridium perfringens; Enterococcus faecalis; Enterococcus faecium; Klebsiella pneumoniae*	4	TP	Partially Concordant	Extra High	SHV; Sul Group; Tet(M); mecA
56	Staphylococcus aureus	*stenotrophomonas maltophilia*	1	TP	UAO	Extra High	Erm(A)/Erm(B); mecA
57	Pseudomonas aeruginosa	*Klebsiella oxytoca*	1	TP	UAO	Extra High	
58	Staphylococcus aureus	*Enterococcus faecalis; Escherichia coli; morganella morganii; proteus mirabilis*	4	TP	UAO	Extra High	CTX-M Group; DHA; Erm(A)/Erm(B); Sul Group; Tet(A)/Tet(B)/Tet(S); Tet(M); dfr Group; mecA
59	Bacteroides fragilis; Klebsiella pneumoniae	*Enterococcus faecium; Staphylococcus lugdunensis*	2	TP	Partially Concordant	Extra High	Erm(A)/Erm(B); SHV; Tet(M); Van Group; mecA
61		*Enterococcus faecalis*	1	FN	Discordant	Extra High	Tet(M); mecA
67	Escherichia coli	*aerococcus urinae; Enterococcus faecalis; Staphylococcus lugdunensis*	3	TP	Partially Concordant	Extra High	Sul Group; Tet(A)/Tet(B)/Tet(S); Tet(M); mecA
68	Candida glabrata	*C. novyi A/B_C. septicum_H. histolytica_P. sordellii group*	4	TP	Partially Concordant	Medium to Low	
69	Candida albicans	*candida glabrata; Enterococcus faecalis*	2	TP	Partially Concordant	Extra High	Erm(A)/Erm(B); Tet(M)
70	Escherichia coli	*Enterococcus faecalis; Staphylococcus aureus*	2	TP	Partially Concordant	Extra High	Tet(M)
71	Staphylococcus aureus	*acinetobacter baumannii; Enterococcus faecalis; stenotrophomonas maltophilia; Streptococcus agalactiae*	4	TP	UAO	Extra High	AAC(6)-Ib (aac(6)-Ib),Ant(3),APH(3)-VIb (aph(3)-VI; Erm(A)/Erm(B); Mef(A); OXA Group; Sul Group; Tet(A)/Tet(B)/Tet(S); Tet(M); mecA
75	Staphylococcus aureus	*acinetobacter baumannii*	1	TP	UAO	Extra High	AAC(6)-Ib (aac(6)-Ib),Ant(3),APH(3)-VIb (aph(3)-VI; OXA Group; Sul Group; Tet(A)/Tet(B)/Tet(S); Tet(M)

Supplemntary Table S5. Cases with PCR only Organisms-continued							
**Case_No**	**Matched PCR and Culture organisms**	**PCR-only (excluding flora & HTC)**	**PCR-only organism**	**Conventional Classification**	**Concordance**	**Ct_16S_Category**	**ABR Gene**
78	Pseudomonas aeruginosa	*Enterococcus faecalis*	1	TP	UAO	Extra High	Erm(A)/Erm(B); Tet(M); mecA
79	Staphylococcus aureus	*Staphylococcus lugdunensis*	1	TP	Partially Concordant	Extra High	Tet(M); mecA
81	Morganella morganii	*Peptostreptococcus anaerobius*	1	TP	Fully Concordant	Extra High	DHA; Erm(A)/Erm(B); Tet(M); mecA
83	Staphylococcus aureus	*Enterococcus faecalis; Peptostreptococcus anaerobius; viridans streptococci*	3	TP	Partially Concordant	Extra High	Sul Group; Tet(M); mecA
89	Staphylococcus aureus	*enterobacter cloacae; pseudomonas aeruginosa*	2	TP	Partially Concordant	Extra High	ampC; mecA
92	Staphylococcus aureus	*Escherichia coli; Peptostreptococcus anaerobius; Streptococcus agalactiae*	3	TP	UAO	Extra High	Erm(A)/Erm(B); Tet(M); mecA
93	Pseudomonas aeruginosa; Staphylococcus aureus; Streptococcus agalactiae	*citrobacter freundii; Enterococcus faecalis; Escherichia coli; Klebsiella oxytoca; Klebsiella pneumoniae*	5	TP	UAO	Extra High	BIL/LAT/CMY; Mef(A); Qnr Group; SHV; Sul Group; Tet(M)
95	Staphylococcus aureus	*enterobacter cloacae; Enterococcus faecalis*	2	TP	UAO	Extra High	Erm(A)/Erm(B); Tet(M); ampC; mecA
98	Staphylococcus aureus	*Enterococcus faecalis; Klebsiella oxytoca*	2	TP	UAO	Extra High	Erm(A)/Erm(B); Tet(M); mecA
101	Pseudomonas aeruginosa	*Enterococcus faecalis; Staphylococcus lugdunensis*	2	TP	Partially Concordant	Extra High	Tet(M); mecA
104	Pseudomonas aeruginosa; Staphylococcus aureus	*Enterococcus faecalis; Klebsiella oxytoca; stenotrophomonas maltophilia; Streptococcus agalactiae*	4	TP	UAO	Extra High	SHV; Sul Group; Tet(M)
105		*Enterococcus faecalis; Staphylococcus lugdunensis*	2	FP	Discordant	High	Tet(M); mecA
106		*Staphylococcus aureus; Staphylococcus lugdunensis*	2	FP	Discordant	Extra High	Tet(M); mecA

Supplementary Table S6. Confirmed S. *aureus, S. agalactiae, P. aeruginosa*-negative cases by culture

Case No	Culture	Normal Flora Organisms (PCR)	CoNS* Matched Culture	PCR organisms (excluding flora & HTC)	S. aureus	S. agalactiae	Ps. aeruginosa
2	skin flora; (S. aureus, S. agalactiae, Ps. aeruginosa: Absent)	CoNS*	True		ND#	ND	ND
5	skin flora; (S. aureus, S. agalactiae, Ps. aeruginosa: Absent)	CoNS	True		ND	ND	ND
11	skin flora; (S. aureus, S. agalactiae, Ps. aeruginosa: Absent)	CoNS	True		ND	ND	ND
13	skin flora; (S. aureus, S. agalactiae, Ps. aeruginosa: Absent)	CoNS	True	Enterococcus faecalis (Ct 28.6); Proteus mirabilis (Ct 28.1)	ND	ND	ND
14	skin flora; (S. aureus, S. agalactiae, Ps. aeruginosa: Absent)	CoNS	True		ND	ND	ND
20	skin flora; (S. aureus, S. agalactiae, Ps. aeruginosa: Absent)	CoNS	True		ND	ND	ND
21	skin flora; (S. aureus, S. agalactiae, Ps. aeruginosa: Absent)	CoNS	True		ND	ND	ND
26	skin flora; (S. aureus, S. agalactiae, Ps. aeruginosa: Absent)	CoNS	True		ND	ND	ND
29	skin flora; (S. aureus, S.	CoNS	True	citrobacter koseri (Ct 19.8);	Detected	ND	ND

	agalactiae, Ps. aeruginosa: Absent)			Enterococcus faecalis (Ct 20.6); Klebsiella oxytoca (Ct 29.5); Staphylococcus aureus (Ct 18.1)			
30	skin flora; (S. aureus, S. agalactiae, Ps. aeruginosa: Absent)	CoNS	True	Staphylococcus lugdunensis (Ct 16.1)	ND	ND	ND
33	skin flora; (S. aureus, S. agalactiae, Ps. aeruginosa: Absent)	CoNS	True	Staphylococcus lugdunensis (Ct 21.9)	ND	ND	ND
35	skin flora; (S. aureus, S. agalactiae, Ps. aeruginosa: Absent)	CoNS	True		ND	ND	ND
37	skin flora; (S. aureus, S. agalactiae, Ps. aeruginosa: Absent)	CoNS	True		ND	ND	ND
43	skin flora; (S. aureus, S. agalactiae, Ps. aeruginosa: Absent)	CoNS	True		ND	ND	ND
44	skin flora; (S. aureus, S. agalactiae, Ps. aeruginosa: Absent)	CoNS	True	Aerococcus urinae (Ct 27.2)	ND	ND	ND
54	skin flora; (S. aureus, S. agalactiae, Ps. aeruginosa: Absent)	CoNS	True	Enterobacter cloacae (Ct 25.7)	ND	ND	ND
60	skin flora; (S. aureus, S. agalactiae, Ps. aeruginosa: Absent)	CoNS	True		ND	ND	ND
63	skin flora; (S. aureus, S. agalactiae, Ps. aeruginosa: Absent)	ND	False		ND	ND	ND
64	skin flora; (S. aureus, S. agalactiae, Ps. aeruginosa: Absent)	CoNS	True		ND	ND	ND

65	skin flora; (S. aureus, S. agalactiae, Ps. aeruginosa: Absent)	CoNS	True		ND	ND	ND
74	skin flora; (S. aureus, S. agalactiae, Ps. aeruginosa: Absent)	ND	False		ND	ND	ND
76	skin flora; (S. aureus, S. agalactiae, Ps. aeruginosa: Absent)	CoNS	True		ND	ND	ND
82	skin flora; (S. aureus, S. agalactiae, Ps. aeruginosa: Absent)	ND	False		ND	ND	ND
90	skin flora; (S. aureus, S. agalactiae, Ps. aeruginosa: Absent)	ND	False		ND	ND	ND
91	skin flora; (S. aureus, S. agalactiae, Ps. aeruginosa: Absent)	CoNS	True		ND	ND	ND
96	skin flora; (S. aureus, S. agalactiae, Ps. aeruginosa: Absent)	CoNS	True		ND	ND	ND
97	skin flora; (S. aureus, S. agalactiae, Ps. aeruginosa: Absent)	ND	False		ND	ND	ND
105	skin flora; (S. aureus, S. agalactiae, Ps. aeruginosa: Absent)	Coagulase-negative Staphylococci species	True	Enterococcus faecalis (Ct 25.9); Staphylococcus lugdunensis (Ct 29.6)	ND	ND	ND

[*] CONS: Coagulase-negative Staphylococci species; [#]ND= Not Detected

Supplementary Table S7. A agalacticae concordance between PCR and Culture													
Case_No	Inclusion_Status	Matched Streptococcus agalactiae	Matched PCR and Culture organisms	PCR organisms (excluding flora & HTC)	Streptococcus agalactiae_Culture Only	*16S rRNA* Ct	Conventional Classification	Concordance	GPC	GPR	GNR	WBC	ABR Gene
6	Included	TRUE	*Streptococcus agalactiae*	*Streptococcus agalactiae (Ct 17.9)*	NA	15.36	TP	Partial TP	High			Present	Tet(M)
12	Included	FALSE	*Staphylococcus aureus*	*Enterococcus faecalis (Ct 18.8); Staphylococcus aureus (Ct 19.2); Streptococcus agalactiae (Ct 31.1)*	Not Detected	16.91	TP	Partial TP	Low		High		Erm(A)/Erm(B); Tet(M); mecA
19	Included	TRUE	*Staphylococcus aureus; Streptococcus agalactiae*	*Enterococcus faecalis (Ct 23.8); Morganella morganii (Ct 26.8); Proteus mirabilis (Ct 22.9); Proteus vulgaris (Ct 27.2); Staphylococcus aureus (Ct 15.3); Streptococcus agalactiae (Ct 19.9)*	NA	13.68	TP	Partial TP	High	High	Low		Erm(A)/Erm(B); Mef(A); Tet(M); mecA
24	Inconclusive	TRUE	*Streptococcus agalactiae*	*Klebsiella pneumoniae (Ct 25.9); Proteus mirabilis (Ct 17.5); Streptococcus agalactiae (Ct 18.4); Viridans streptococci (Ct 17.7)*	NA	12.91	Inconclusive	Inconclusive	High	Medium	High		Erm(A)/Erm(B); Mef(A); SHV; Tet(M)
25	Included	TRUE	*Staphylococcus aureus; Streptococcus agalactiae*	*Enterobacter cloacae (Ct 18.1); Enterococcus faecalis (Ct 20.7); Staphylococcus aureus (Ct 24.4); Streptococcus agalactiae (Ct 23.2); Viridans streptococci (Ct 22.5)*	NA	14.98	TP	TP UAO	High		Low		AAC(6)-Ib (aac(6)-Ib),Ant(3),APH(3)-VIb (aph(3)-VI; Erm(A)/Erm(B); Mef(A); Sul Group; Tet(M); ampC; mecA
34	Included	TRUE	*Streptococcus agalactiae*	*Streptococcus agalactiae (Ct 19.0)*	NA	15.36	TP	TP	High	Low			Erm(A)/Erm(B); Tet(M); mecA
36	Included	FALSE	*Pseudomonas aeruginosa; Staphylococcus aureus*	*Peptostreptococcus anaerobius (Ct 25.6); Pseudomonas aeruginosa (Ct 20.4); Staphylococcus aureus (Ct 19.0); Staphylococcus lugdunensis (Ct 31.2); Streptococcus agalactiae (Ct 27.7); Viridans streptococci (Ct 26.2)*	Not Detected	14.9	TP	Partial TP	Low		High		Erm(A)/Erm(B); Mef(A); Tet(M); mecA

Supplementary Table S7. A agalacticae concordance between PCR and Culture-continued													
Case_No	Inclusion_Status	Matched Streptococcus agalactiae	Matched PCR and Culture organisms	PCR organisms (excluding flora & HTC)	Streptococcus agalactiae_Culture Only	*16S rRNA* Ct	Conventional Classification	Concordance	GPC	GPR	GNR	WBC	ABR Gene
71	Included	FALSE	*Staphylococcus aureus*	*acinetobacter baumannii (Ct 19.9); Enterococcus faecalis (Ct 24.4); Staphylococcus aureus (Ct 18.7); Stenotrophomonas maltophilia (Ct 19.2); Streptococcus agalactiae (Ct 32.2)*	Not Detected	14.37	TP	TP UAO	High	High		Present	AAC(6)-Ib (aac(6)-Ib),Ant(3),APH(3)-VIb (aph(3)-VI; Erm(A)/Erm(B); Mef(A); OXA Group; Sul Group; Tet(A)/Tet(B)/Tet(S); Tet(M); mecA
92	Included	FALSE	*Staphylococcus aureus*	*Escherichia coli (Ct 27.3); Peptostreptococcus anaerobius (Ct 33.1); Staphylococcus aureus (Ct 27.0); Streptococcus agalactiae (Ct 25.4)*	Not Detected	22.51	TP	TP UAO	High				Erm(A)/Erm(B); Tet(M); mecA
93	Included	TRUE	*Pseudomonas aeruginosa; Staphylococcus aureus; Streptococcus agalactiae*	*citrobacter freundii (Ct 17.7); Enterococcus faecalis (Ct 27.2); Escherichia coli (Ct 24.6); Klebsiella oxytoca (Ct 22.4); Klebsiella pneumoniae (Ct 19.8); Pseudomonas aeruginosa (Ct 26.3); Staphylococcus aureus (Ct 30.1); Streptococcus agalactiae (Ct 28.9)*	NA	14.96	TP	TP UAO	Low		High		BIL/LAT/CMY; Mef(A); Qnr Group; SHV; Sul Group; Tet(M)
104	Included	FALSE	*Pseudomonas aeruginosa; Staphylococcus aureus*	*Enterococcus faecalis (Ct 30.9); Klebsiella oxytoca (Ct 18.7); Pseudomonas aeruginosa (Ct 17.3); Staphylococcus aureus (Ct 30.6); Stenotrophomonas maltophilia (Ct 25.0); Streptococcus agalactiae (Ct 30.4)*	Not Detected	14.25	TP	TP UAO			High		SHV; Sul Group; Tet(M)

Supplementary Table S8.Gram stain- GNR Positive cases

No	Case_No	Inclusion_Status	Matched PCR and Culture organisms	PCR organisms (excluding flora & HTC)	GNR Present in Culture	GNR Present in PCR	Ct_16S	Conventional Classification	Concordance
1	1	Included	*Escherichia coli*	*Enterobacter cloacae (Ct 33.0); Enterococcus faecium (Ct 31.3); Escherichia coli (Ct 16.8)*	TRUE	TRUE	14.8	TP	Partially Concordant
2	4	Included	*Pseudomonas aeruginosa*	*Pseudomonas aeruginosa (Ct 20.7)*	TRUE	TRUE	17.86	TP	Fully Concordant
3	12	Included	*Staphylococcus aureus*	*Enterococcus faecalis (Ct 18.8); Staphylococcus aureus (Ct 19.2); Streptococcus agalactiae (Ct 31.1)*	FALSE	FALSE	16.91	TP	Partially Concordant
4	15	Included	*Staphylococcus aureus*	*Enterococcus faecalis (Ct 17.9); Proteus mirabilis (Ct 30.3); Staphylococcus aureus (Ct 16.2)*	FALSE	TRUE	14.13	TP	Partially Concordant
5	16	Included	*Staphylococcus aureus*	*Staphylococcus aureus (Ct 19.2)*	FALSE	FALSE	17.84	TP	Fully Concordant
6	18	Included	*Pseudomonas aeruginosa*	*Enterococcus faecalis (Ct 19.4); Pseudomonas aeruginosa (Ct 20.4)*	TRUE	TRUE	14.81	TP	Partially Concordant
7	19	Included	*Staphylococcus aureus; Streptococcus agalactiae*	*Enterococcus faecalis (Ct 23.8); Morganella morganii (Ct 26.8); Proteus mirabilis (Ct 22.9); Proteus vulgaris (Ct 27.2); Staphylococcus aureus (Ct 15.3); Streptococcus agalactiae (Ct 19.9)*	FALSE	TRUE	13.68	TP	Partially Concordant
8	21	Included			FALSE	FALSE	30.75	TN	Fully Concordant
9	22	Included	*Staphylococcus aureus*	*Enterococcus faecalis (Ct 23.2); Staphylococcus aureus (Ct 17.1)*	FALSE	FALSE	14.13	TP	Partially Concordant
10	23	Exclude (Mixed results)		Streptococcus pyogenes (Ct 34.0)	Excluded (MIXED)	Excluded (MIXED)	27.72	Exclude (Mixed results)	Exclude (Unidentified)
11	24	Inconclusive culture	Streptococcus agalactiae	Klebsiella pneumoniae (Ct 25.9); Proteus mirabilis (Ct 17.5); Streptococcus agalactiae (Ct 18.4); Viridans streptococci (Ct 17.7)	Inconclusive culture	TRUE	12.91	Inconclusive culture	Inconclusive culture
12	25	Included	*Staphylococcus aureus; Streptococcus agalactiae*	*Enterobacter cloacae (Ct 18.1); Enterococcus faecalis (Ct 20.7); Staphylococcus aureus (Ct 24.4); Streptococcus agalactiae (Ct 23.2); Viridans streptococci (Ct 22.5)*	UAO	TRUE	14.98	TP	TP UAO

Supplementary Table S8.Gram stain- GNR Positive cases-continued

No	Case_No	Inclusion_Status	Matched PCR and Culture organisms	PCR organisms (excluding flora & HTC)	GNR Present in Culture	GNR Present in PCR	Ct_16S	Conventional Classification	Concordance
13	28	Included	*Staphylococcus aureus; Bacteroides fragilis*	*Bacteroides fragilis (Ct 21.9); Enterococcus faecalis (Ct 23.8); Staphylococcus aureus (Ct 18.7)*	UAO	TRUE	15.62	TP	Partially Concordant
14	29	Included		*citrobacter koseri (Ct 19.8); Enterococcus faecalis (Ct 20.6); Klebsiella oxytoca (Ct 29.5); Staphylococcus aureus (Ct 18.1)*	UAO	TRUE	13.99	FP	Discordant
15	36	Included	*Pseudomonas aeruginosa; Staphylococcus aureus*	*peptoStreptococcus anaerobius (Ct 25.6); Pseudomonas aeruginosa (Ct 20.4); Staphylococcus aureus (Ct 19.0); Staphylococcus lugdunensis (Ct 31.2); Streptococcus agalactiae (Ct 27.7); Viridans streptococci (Ct 26.2)*	TRUE	TRUE	14.9	TP	Partially Concordant
16	40	Inconclusive culture		Enterococcus faecalis (Ct 27.2); Escherichia coli (Ct 19.0)	Inconclusive culture	TRUE	16.49	Inconclusive culture	Inconclusive culture
17	41	Included	*Staphylococcus aureus*	*Enterococcus faecalis (Ct 19.6); Klebsiella pneumoniae (Ct 19.6); Staphylococcus aureus (Ct 20.0); Viridans streptococci (Ct 28.7)*	FALSE	TRUE	15.07	TP	Partially Concordant
18	42	Included	*Serratia marcescens*	*Serratia marcescens (Ct 16.0)*	TRUE	TRUE	14.09	TP	Partially Concordant
19	45	Included	*Staphylococcus aureus*	*Klebsiella pneumoniae (Ct 23.7); Staphylococcus aureus (Ct 19.0)*	FALSE	TRUE	15.34	TP	TP UAO
20	46	Included	*Pseudomonas aeruginosa; Bacteroides fragilis*	*Bacteroides fragilis (Ct 20.4); Citrobacter koseri (Ct 27.8); Morganella morganii (Ct 15.1); Pseudomonas aeruginosa (Ct 18.8); Serratia marcescens (Ct 19.9)*	TRUE	TRUE	14.35	TP	TP UAO
21	47	Included	*Pseudomonas aeruginosa*	*Enterococcus faecalis (Ct 22.4); Proteus mirabilis (Ct 18.0); Pseudomonas aeruginosa (Ct 17.6)*	TRUE	TRUE	15.16	TP	Partially Concordant
22	48	Included	*Staphylococcus aureus*	*Enterococcus faecalis (Ct 23.52); Staphylococcus aureus (Ct 20.5); Stenotrophomonas maltophilia (Ct 31.3); Viridans streptococci (Ct 24)*	FALSE	TRUE	15.75	TP	Partially Concordant

Supplementary Table S8.Gram stain- GNR Positive cases-continued

No	Case_No	Inclusion_Status	Matched PCR and Culture organisms	PCR organisms (excluding flora & HTC)	GNR Present in Culture	GNR Present in PCR	Ct_16S	Conventional Classification	Concordance
23	49	Included	*Escherichia coli*	*Escherichia coli (Ct 17.4); Staphylococcus lugdunensis (Ct 17.7)*	TRUE	TRUE	13.96	TP	Partially Concordant
24	50	Inconclusive culture		Enterococcus faecalis (Ct 22.7); Klebsiella oxytoca (Ct 18.8); Serratia marcescens (Ct 14.9)	Inconclusive culture	TRUE	12.66	Inconclusive culture	Inconclusive culture
25	52	Included	*Pseudomonas aeruginosa*	*Enterobacter cloacae (Ct 24.2); Enterococcus faecalis (Ct 23.5); Klebsiella pneumoniae (Ct 26.9); Pseudomonas aeruginosa (Ct 21.7)*	TRUE	TRUE	14.75	TP	TP UAO
26	55	Included	*Staphylococcus aureus*	*Clostridium perfringens (Ct 25.2); Enterococcus faecalis (Ct 18.3); Enterococcus faecium (Ct 27.2); Klebsiella pneumoniae (Ct 16.5); Staphylococcus aureus (Ct 22.9)*	FALSE	TRUE	13.9	TP	Partially Concordant
27	56	Included	*Staphylococcus aureus*	*Staphylococcus aureus (Ct 20.9); Stenotrophomonas maltophilia (Ct 26.5)*	FALSE	TRUE	18.4	TP	TP UAO
28	57	Included	*Pseudomonas aeruginosa*	*Klebsiella oxytoca (Ct 29.8); Pseudomonas aeruginosa (Ct 19.7)*	TRUE	TRUE	17.08	TP	TP UAO
29	58	Included	*Staphylococcus aureus*	*Enterococcus faecalis (Ct 27.9); Escherichia coli (Ct 24.0); Morganella morganii (Ct 17.8); Proteus mirabilis (Ct 20.0); Staphylococcus aureus (Ct 21.8)*	FALSE	TRUE	15.15	TP	TP UAO
30	59	Included	*Bacteroides fragilis; Klebsiella pneumoniae*	*Bacteroides fragilis (Ct 21.9); Enterococcus faecium (Ct 30.9); Klebsiella pneumoniae (Ct 19.9); Staphylococcus lugdunensis (Ct 27.3)*	TRUE	TRUE	16.83	TP	Partially Concordant
31	62	Included	*Pseudomonas aeruginosa*	*Pseudomonas aeruginosa (Ct 21.1)*	TRUE	TRUE	17.78	TP	Fully Concordant
32	66	Included			FALSE	FALSE	15.7	TN	Fully Concordant

Supplementary Table S8.Gram stain- GNR Positive cases-continued

No	Case_No	Inclusion_Status	Matched PCR and Culture organisms	PCR organisms (excluding flora & HTC)	GNR Present in Culture	GNR Present in PCR	Ct_16S	Conventional Classification	Concordance
33	67	Included	*Escherichia coli*	*Aerococcus urinae (Ct 33.34584); Enterococcus faecalis (Ct 26.32332); Escherichia coli (Ct 18.65653); Staphylococcus lugdunensis (Ct 30.55972)*	TRUE	FALSE	14.74	TP	Partially Concordant
34	70	Included	*Escherichia coli*	*Enterococcus faecalis (Ct 22.5); Escherichia coli (Ct 18.1); Staphylococcus aureus (Ct 30.9)*	TRUE	TRUE	15.95	TP	Partially Concordant
35	72	Inconclusive culture		Staphylococcus lugdunensis (Ct 23.8)	Inconclusive culture	FALSE	20.56	Inconclusive culture	Inconclusive culture
36	73	Included	*Pseudomonas aeruginosa*	*Pseudomonas aeruginosa (Ct 21.0)*	TRUE	TRUE	18.03	TP	Fully Concordant
37	77	Inconclusive culture		Enterobacter cloacae (Ct 18.5); Enterococcus faecalis (Ct 29.9); Klebsiella oxytoca (Ct 24.6)	Inconclusive culture	TRUE	15.53	Inconclusive culture	Inconclusive culture
38	78	Included	*Pseudomonas aeruginosa*	*Enterococcus faecalis (Ct 18.0); Pseudomonas aeruginosa (Ct 20.5)*	TRUE	TRUE	16.21	TP	TP UAO
39	83	Included	*Staphylococcus aureus*	*Enterococcus faecalis (Ct 23.7); peptoStreptococcus anaerobius (Ct 27.7); Staphylococcus aureus (Ct 21.4); Viridans streptococci (Ct 27.2)*	FALSE	FALSE	20.67	TP	Partially Concordant
40	85	Inconclusive culture		Viridans streptococci (Ct 25.4)	Inconclusive culture	FALSE	24.33	Inconclusive culture	Inconclusive culture
41	89	Included	*Staphylococcus aureus*	*Enterobacter cloacae (Ct 18.0); Pseudomonas aeruginosa (Ct 26.5); Staphylococcus aureus (Ct 20.7)*	FALSE	TRUE	14.78	TP	Partially Concordant
42	90	Included			FALSE	FALSE	23.12	TN	Fully Concordant
43	93	Included	*Pseudomonas aeruginosa; Staphylococcus aureus; Streptococcus agalactiae*	*Citrobacter freundii (Ct 17.7); Enterococcus faecalis (Ct 27.2); Escherichia coli (Ct 24.6); Klebsiella oxytoca (Ct 22.4); Klebsiella pneumoniae (Ct 19.8); Pseudomonas aeruginosa (Ct 26.3); Staphylococcus aureus (Ct 30.1); Streptococcus agalactiae (Ct 28.9)*	TRUE	TRUE	14.96	TP	TP UAO

Supplementary Table S8.Gram stain- GNR Positive cases-continued

No	Case_No	Inclusion_Status	Matched PCR and Culture organisms	PCR organisms (excluding flora & HTC)	GNR Present in Culture	GNR Present in PCR	Ct_16S	Conventional Classification	Concordance
44	95	Included	*Staphylococcus aureus*	*Enterobacter cloacae (Ct 17.3); Enterococcus faecalis (Ct 26.6); Staphylococcus aureus (Ct 22.2)*	FALSE	TRUE	14.62	TP	TP UAO
45	98	Included	*Staphylococcus aureus*	*Enterococcus faecalis (Ct 23.1); Klebsiella oxytoca (Ct 26.8); Staphylococcus aureus (Ct 26.5)*	FALSE	TRUE	21.18	TP	TP UAO
46	102	Inconclusive culture		Enterococcus faecalis (Ct 19.2); Proteus mirabilis (Ct 19.3); Staphylococcus saprophyticus (Ct 35.5)	Inconclusive culture	TRUE	16.15	Inconclusive culture	Inconclusive culture
47	104	Included	*Pseudomonas aeruginosa; Staphylococcus aureus*	*Enterococcus faecalis (Ct 30.9); Klebsiella oxytoca (Ct 18.7); Pseudomonas aeruginosa (Ct 17.3); Staphylococcus aureus (Ct 30.6); Stenotrophomonas maltophilia (Ct 25.0); Streptococcus agalactiae (Ct 30.4)*	TRUE	TRUE	14.25	TP	TP UAO

Supplementary Table S9. Logistic Regression Models Integrating Ct16S and Gram Stain terms

Model	AUC	Ct_16S OR	Ct_16S p value	Gram Odd Ratio	Gram p value
Ct16S + Gram_Score_Max	0.842	0.794	0.0014	0.957	0.8985
Ct16S + Gram_Score_Sum	0.862	0.84	0.0066	1.317	0.2096
Ct16S + Gram_Score_GNRPriority	0.842	0.79	0.0013	0.923	0.8147
Ct16S only	0.847	0.799	0.0	N/A	N/A
Ct16S + GPC_binary	0.857	0.786	0.0001	0.612	0.5319
Ct16S + GNR_binary	0.868	0.837	0.0015	6.545	0.0261
Ct16S + GPC_GNR_binary	0.848	0.795	0.0008	0.898	0.9178

Supplementary Table S10. Predicted Infection Probability for False Positive Cases
Predicted probability of true infection for nine PCR-positive, culture-negative wound specimens classified as false positives under conventional diagnostic criteria. All cases exhibited high bacterial burden (Ct ≤ 24.4), and eight of nine had Gram-positive cocci observed on Gram stain. Probabilities were derived from the trained logistic model using 16S rRNA Ct and sum of of the Gram morphology scores (GNR & GPC). Calibration was done using 5-fold Platt scaling via CalibratedClassifierCV(method="sigmoid"). The AUC value on training set was 0.862.

Case No	Ct_16S	PCR Organisms	Gram Stain & culture note	Predicted Infection Probability %
30	13.7	*Coagulase-negative Staphylococci species (Ct 15.9); Finegoldia magna (Ct 25.09); Staphylococcus lugdunensis (Ct 16.09)*	GPC: High; Skin Flora	88.2%
29	14.0	*citrobacter koseri (Ct 19.8); Enterococcus faecalis (Ct 20.6); Klebsiella oxytoca (Ct 29.5); Staphylococcus aureus (Ct 18.1)*	GPC: High; GNR: Low; GPR: High; Skin Flora;	90.03%
33	15.0	*Coagulase-negative Staphylococci species (Ct 17.6); Finegoldia magna (Ct 21.21); Staphylococcus lugdunensis (Ct 21.91)*	GPC: High; GPR: Medium; Skin Flora	86.0%
44	15.7	*Aerococcus urinae (Ct 27.16); Coagulase-negative Staphylococci species (Ct 18.2); Finegoldia magna (Ct 23.8); Peptoniphilus Species (Ct 24.2)*	GPC: Medium; Skin Flora	80.8%
106	16.0	*Coagulase-negative Staphylococci species (Ct 33.67); Finegoldia magna (Ct 30.25); Peptoniphilus Species (Ct 30.7); Staphylococcus aureus (Ct 32.27); Staphylococcus lugdunensis (Ct 32.03)*	GPR: High; Skin Flora	70.05%
13	20.5	*Coagulase-negative Staphylococci species (Ct 27.14); Enterococcus faecalis (Ct 28.62); Proteus mirabilis (Ct 28.12)*	GPC: High: Skin Flora	72.3%
54	21.1	*Coagulase-negative Staphylococci species (Ct 31.45); Enterobacter cloacae (Ct 25.73)*	GPC: High: Skin Flora	70.2%
7	22.1	*Enterococcus faecalis (Ct 22.64)*	WBC: Present	48.02%
105	24.4	*Coagulase-negative Staphylococci species (Ct 26.7); Enterococcus faecalis (Ct 25.90); Staphylococcus lugdunensis (Ct 29.64)*	GPC: High: Skin Flora	58.62%

Supplementary Table S11. All PCR organisms' probabilities

No.	Case_No	Organism	Ct	Ct_16S	GPC	GPR	GNR	Is Culture Positive?	Calibrated_Prob
1	1	*E. cloacae*	33	14.8	Low	Absent	High	FALSE	0.636
2	1	*E. faecium*	31.3	14.8	Low	Absent	High	FALSE	0.717
3	1	*E. coli*	16.8	14.8	Low	Absent	High	TRUE	0.980
4	3	*S. aureus*	19.2	16.71	High	Absent	Absent	TRUE	0.960
5	4	*P. aeruginosa*	20.7	17.86	Absent	Absent	High	TRUE	0.927
6	6	*S. agalactiae*	17.9	15.36	High	Absent	Absent	TRUE	0.976
7	7	*E. faecalis*	22.6	22.07	Absent	Absent	Absent	FALSE	0.807
8	8	*E. faecalis*	30.7	26.32	Absent	Absent	Absent	FALSE	0.285
9	10	*S. aureus*	28.9	23.74	High	Absent	Absent	TRUE	0.506
10	12	*S. aureus*	19.2	16.91	Low	Absent	High	TRUE	0.955
11	12	*S. agalactiae*	31.1	16.91	Low	Absent	High	FALSE	0.651
12	12	*E. faecalis*	18.8	16.91	Low	Absent	High	FALSE	0.958
13	13	*E. faecalis*	28.6	20.48	High	Absent	Absent	FALSE	0.651
14	13	*P. mirabilis*	28.1	20.48	High	Absent	Absent	FALSE	0.643
15	15	*S. aureus*	16.2	14.13	High	Absent	High	TRUE	0.986
16	15	*E. faecalis*	17.9	14.13	High	Absent	High	FALSE	0.980
17	15	*P. mirabilis*	30.3	14.13	High	Absent	High	FALSE	0.769
18	16	*S. aureus*	19.2	17.84	High	Absent	Low	TRUE	0.952
19	17	*S. aureus*	17.1	14.48	High	Absent	Absent	TRUE	0.982
20	18	*P. aeruginosa*	20.4	14.81	High	Absent	High	TRUE	0.958
21	18	*E. faecalis*	19.4	14.81	High	Absent	High	FALSE	0.970
22	19	*S. aureus*	15.3	13.68	High	High	Low	TRUE	0.989
23	19	*S. agalactiae*	19.9	13.68	High	High	Low	TRUE	0.972
24	19	*E. faecalis*	23.8	13.68	High	High	Low	FALSE	0.940
25	19	*P. mirabilis*	22.9	13.68	High	High	Low	FALSE	0.942
26	19	*M. morganii*	26.8	13.68	High	High	Low	FALSE	0.879
27	19	*P. vulgaris*	27.2	13.68	High	High	Low	FALSE	0.870
28	22	*S. aureus*	17.1	14.13	High	High	High	TRUE	0.983
29	22	*E. faecalis*	23.2	14.13	High	High	High	FALSE	0.942
30	25	*E. cloacae*	18.1	14.98	High	Absent	Low	FALSE	0.973
31	25	*S. aureus*	24.4	14.98	High	Absent	Low	TRUE	0.917
32	25	*S. agalactiae*	23.2	14.98	High	Absent	Low	TRUE	0.934
33	25	*E. faecalis*	20.7	14.98	High	Absent	Low	FALSE	0.960
34	25	*Viridians streptococ*	22.5	14.98	High	Absent	Low	FALSE	0.942
35	27	*S. aureus*	18.8	16.18	High	Absent	Absent	TRUE	0.966
36	28	*S. aureus*	18.7	15.62	High	Absent	High	TRUE	0.970

37	28	*E. faecalis*	23.8	15.62	High	Absent	High	FALSE	0.918
38	28	*B. fragilis*	21.9	15.62	High	Absent	High	TRUE	0.935
39	29	*S. aureus*	18.1	13.99	High	High	Low	FALSE	0.980
40	29	*E. faecalis*	20.6	13.99	High	High	Low	FALSE	0.966
41	29	*c. koseri*	19.8	13.99	High	High	Low	FALSE	0.967
42	29	*K. oxytoca*	29.5	13.99	High	High	Low	FALSE	0.800
43	30	*S. lugdunensis*	16.1	13.71	High	Absent	Absent	FALSE	0.987
44	33	*S. lugdunensis*	21.9	15.02	High	Medium	Absent	FALSE	0.948
45	34	*S. agalactiae*	19	15.36	High	Low	Absent	TRUE	0.969
46	36	*S. aureus*	19	14.9	Low	Absent	High	TRUE	0.969
47	36	*P. aeruginosa*	20.4	14.9	Low	Absent	High	TRUE	0.957
48	36	*S. agalactiae*	27.7	14.9	Low	Absent	High	FALSE	0.838
49	36	*V. streptococci*	26.2	14.9	Low	Absent	High	FALSE	0.875
50	36	*S. lugdunensis*	31.2	14.9	Low	Absent	High	FALSE	0.718
51	36	*P. anaerobius*	25.6	14.9	Low	Absent	High	FALSE	0.883
52	39	*S. aureus*	17	16	High	Absent	Absent	TRUE	0.977
53	41	*S. aureus*	20	15.07	High	High	High	TRUE	0.964
54	41	*E. faecalis*	19.6	15.07	High	High	High	FALSE	0.967
55	41	*V. streptococci*	28.7	15.07	High	High	High	FALSE	0.818
56	41	*K. pneumoniae*	19.6	15.07	High	High	High	FALSE	0.962
57	42	*S. marcescens*	16	14.09	Absent	Absent	High	TRUE	0.985
58	44	*A. urinae*	27.2	15.73	Medium	Absent	Absent	FALSE	0.826
59	45	*S. aureus*	19	15.34	High	Absent	High	TRUE	0.970
60	45	*K. pneumoniae*	23.7	15.34	High	Absent	High	FALSE	0.913
61	46	*P. aeruginosa*	18.8	14.4	Medium	Absent	High	TRUE	0.971
62	46	*M. morganii*	15.1	14.4	Medium	Absent	High	FALSE	0.987
63	46	*B. fragilis*	20.4	14.4	Medium	Absent	High	TRUE	0.961
64	46	*S. marcescens*	19.9	14.4	Medium	Absent	High	FALSE	0.964
65	46	*C. koseri*	27.8	14.4	Medium	Absent	High	FALSE	0.841
66	47	*P. aeruginosa*	17.6	15.16	Low	Absent	High	TRUE	0.975
67	47	*E. faecalis*	22.4	15.16	Low	Absent	High	FALSE	0.936
68	47	*P. mirabilis*	18	15.16	Low	Absent	High	FALSE	0.972
69	48	*S. aureus*	20.5	15.75	High	Medium	High	TRUE	0.956
70	48	*E. faecalis*	23.52	15.75	High	Medium	High	FALSE	0.920
71	48	*V. streptococci*	24	15.75	High	Medium	High	FALSE	0.913
72	48	*S. maltophilia*	31.3	15.75	High	Medium	High	FALSE	0.677
73	49	*E. coli*	17.4	13.96	High	Absent	High	TRUE	0.980
74	49	*S. lugdunensis*	17.7	13.96	High	Absent	High	FALSE	0.981

75	52	*E. cloacae*	24.2	14.75	Low	Absent	High	FALSE	0.912
76	52	*P. aeruginosa*	21.7	14.75	Low	Absent	High	TRUE	0.946
77	52	*E. faecalis*	23.5	14.75	Low	Absent	High	FALSE	0.926
78	52	*K. pneumoniae*	26.9	14.75	Low	Absent	High	FALSE	0.857
79	54	*E. cloacae*	25.7	21.14	High	Absent	Absent	FALSE	0.723
80	55	*E. faecium*	27.2	13.9	High	Low	High	FALSE	0.881
81	55	*S. aureus*	22.9	13.9	High	Low	High	TRUE	0.948
82	55	*E. faecalis*	18.3	13.9	High	Low	High	FALSE	0.979
83	55	*K. pneumoniae*	16.5	13.9	High	Low	High	FALSE	0.984
84	55	*C. perfringens*	25.2	13.9	High	Low	High	FALSE	0.907
85	56	*S. aureus*	20.9	18.4	High	Absent	High	TRUE	0.927
86	56	*S. maltophilia*	26.5	18.4	High	Absent	High	FALSE	0.779
87	57	*P. aeruginosa*	19.7	17.08	Absent	Low	High	TRUE	0.947
88	57	*K. oxytoca*	29.8	17.08	Absent	Low	High	FALSE	0.694
89	58	*E. coli*	24	15.15	Medium	Absent	High	FALSE	0.910
90	58	*S. aureus*	21.8	15.15	Medium	Absent	High	TRUE	0.941
91	58	*E. faecalis*	27.9	15.15	Medium	Absent	High	FALSE	0.819
92	58	*P. mirabilis*	20	15.15	Medium	Absent	High	FALSE	0.959
93	58	*M. morganii*	17.8	15.15	Medium	Absent	High	FALSE	0.974
94	59	*E. faecium*	30.9	16.83	Low	Absent	High	FALSE	0.663
95	59	*B. fragilis*	21.9	16.83	Low	Absent	High	TRUE	0.922
96	59	*S. lugdunensis*	27.3	16.83	Low	Absent	High	FALSE	0.802
97	59	*K. pneumoniae*	19.9	16.83	Low	Absent	High	TRUE	0.947
98	61	*E. faecalis*	21.6	20.14	High	Absent	Absent	FALSE	0.891
99	62	*P. aeruginosa*	21.1	17.78	High	Absent	High	TRUE	0.922
100	67	*E. coli*	18.65653	14.74	High	Absent	High	TRUE	0.971
101	67	*E. faecalis*	26.32332	14.74	High	Absent	High	FALSE	0.885
102	67	*S. lugdunensis*	30.55972	14.74	High	Absent	High	FALSE	0.765
103	67	*A. urinae*	33.34584	14.74	High	Absent	High	FALSE	0.651
104	68	*C. novyi A/B group*	32	31.19	Absent	Absent	Absent	FALSE	0.110
105	69	*E. faecalis*	21.1	19.88	High	Absent	Absent	FALSE	0.904
106	70	*E. coli*	18.1	15.95	Medium	Absent	High	TRUE	0.968
107	70	*S. aureus*	30.9	15.95	Medium	Absent	High	FALSE	0.685
108	70	*E. faecalis*	22.5	15.95	Medium	Absent	High	FALSE	0.923
109	71	*S. aureus*	18.7	14.37	High	High	Absent	TRUE	0.976
110	71	*S. agalactiae*	32.2	14.37	High	High	Absent	FALSE	0.714
111	71	*E. faecalis*	24.4	14.37	High	High	Absent	FALSE	0.924
112	71	*S. maltophilia*	19.2	14.37	High	High	Absent	FALSE	0.969

113	71	*a. baumannii*	19.9	14.37	High	High	Absent	FALSE	0.964
114	73	*P. aeruginosa*	21	18.03	Absent	Absent	High	TRUE	0.920
115	75	*S. aureus*	18.6	15.51	Absent	High	Absent	TRUE	0.967
116	75	*a. baumannii*	26.1	15.51	Absent	High	Absent	FALSE	0.860
117	78	*P. aeruginosa*	20.5	16.21	High	Absent	High	TRUE	0.946
118	78	*E. faecalis*	18	16.21	High	Absent	High	FALSE	0.971
119	79	*S. aureus*	21.8	21.57	High	Absent	Absent	TRUE	0.860
120	79	*S. lugdunensis*	32.8	21.57	High	Absent	Absent	FALSE	0.401
121	81	*M. morganii*	16.1	15.13	Low	Absent	Absent	TRUE	0.981
122	81	*P. anaerobius*	27	15.13	Low	Absent	Absent	FALSE	0.845
123	83	*S. aureus*	21.4	20.67	High	Absent	Medium	TRUE	0.886
124	83	*E. faecalis*	23.7	20.67	High	Absent	Medium	FALSE	0.829
125	83	*V. streptococci*	27.2	20.67	High	Absent	Medium	FALSE	0.705
126	83	*P. anaerobius*	27.7	20.67	High	Absent	Medium	FALSE	0.654
127	84	*S. aureus*	22.4	19.99	High	Absent	Absent	TRUE	0.877
128	86	*S. aureus*	24.7	19.13	Low	Absent	Absent	TRUE	0.824
129	88	*S. aureus*	26.4	24.43	Low	Absent	Absent	TRUE	0.580
130	89	*E. cloacae*	18	14.78	High	Absent	High	FALSE	0.974
131	89	*S. aureus*	20.7	14.78	High	Absent	High	TRUE	0.961
132	89	*P. aeruginosa*	26.5	14.78	High	Absent	High	FALSE	0.866
133	92	*E. coli*	27.3	22.51	High	Absent	Absent	FALSE	0.602
134	92	*S. aureus*	27	22.51	High	Absent	Absent	TRUE	0.648
135	92	*S. agalactiae*	25.4	22.51	High	Absent	Absent	FALSE	0.717
136	92	*P. anaerobius*	33.1	22.51	High	Absent	Absent	FALSE	0.319
137	93	*E. coli*	24.6	14.96	Low	Absent	High	FALSE	0.903
138	93	*S. aureus*	30.1	14.96	Low	Absent	High	TRUE	0.759
139	93	*P. aeruginosa*	26.3	14.96	Low	Absent	High	TRUE	0.867
140	93	*S. agalactiae*	28.9	14.96	Low	Absent	High	TRUE	0.800
141	93	*E. faecalis*	27.2	14.96	Low	Absent	High	FALSE	0.850
142	93	*K. oxytoca*	22.4	14.96	Low	Absent	High	FALSE	0.936
143	93	*K. pneumoniae*	19.8	14.96	Low	Absent	High	FALSE	0.962
144	93	*c. freundii*	17.7	14.96	Low	Absent	High	FALSE	0.975
145	95	*E. cloacae*	17.3	14.62	High	Low	High	FALSE	0.978
146	95	*S. aureus*	22.2	14.62	High	Low	High	TRUE	0.949
147	95	*E. faecalis*	26.6	14.62	High	Low	High	FALSE	0.881
148	98	*S. aureus*	26.5	21.18	Medium	Low	Low	TRUE	0.689
149	98	*E. faecalis*	23.1	21.18	Medium	Low	Low	FALSE	0.814
150	98	*K. oxytoca*	26.8	21.18	Medium	Low	Low	FALSE	0.676

151	99	*S. lugdunensis*	28.3	26.96	Low	Absent	Absent	TRUE	0.381
152	100	*S. aureus*	31.3	29.75	High	Absent	Absent	TRUE	0.178
153	101	*P. aeruginosa*	16.7	14.25	Absent	High	Absent	TRUE	0.982
154	101	*E. faecalis*	18.7	14.25	Absent	High	Absent	FALSE	0.973
155	101	*S. lugdunensis*	24.5	14.25	Absent	High	Absent	FALSE	0.914
156	103	*S. aureus*	16.9	14.33	High	Absent	Absent	TRUE	0.983
157	104	*S. aureus*	30.6	14.25	Absent	Absent	High	TRUE	0.754
158	104	*P. aeruginosa*	17.3	14.25	Absent	Absent	High	TRUE	0.980
159	104	*S. agalactiae*	30.4	14.25	Absent	Absent	High	FALSE	0.761
160	104	*E. faecalis*	30.9	14.25	Absent	Absent	High	FALSE	0.742
161	104	*K. oxytoca*	18.7	14.25	Absent	Absent	High	FALSE	0.973
162	104	*S. maltophilia*	25	14.25	Absent	Absent	High	FALSE	0.906
163	105	*E. faecalis*	25.9	24.4	High	Absent	Absent	FALSE	0.626
164	105	*S. lugdunensis*	29.6	24.4	High	Absent	Absent	FALSE	0.443
165	106	*S. aureus*	32.3	15.97	Absent	High	Absent	FALSE	0.622
166	106	*S. lugdunensis*	32	15.97	Absent	High	Absent	FALSE	0.635
167	107	*S. aureus*	16.1	13.67	High	Absent	Absent	TRUE	0.987

Supplementary Table S12. Organism-level diagnostic performance metrics for PCR and culture using a probability-adjusted symmetric framework, alongside comparative PCR performance using culture as a reference. Each organism is represented by paired rows for culture and PCR methods. Diagnostic metrics include true positives (TP), false positives (FP), false negatives (FN), true negatives (TN), and calculated sensitivity and specificity under a probability-informed symmetric classification framework. For PCR, performance metrics based on treating culture as the reference standard are included for comparison**.**

Organism	Method	TP	FP	FN	TN	PA_Sensitivity	PA_Specificity	CR Sensitivity	CR Specificity
A. baumannii	Culture	0	0	2	69	0.0	1.0	n/a	0.972
	PCR	2	0	0	69	1.0	1.0	0.972	0.972
A. urinae	Culture	0	0	1	70	0.0	1.0	n/a	0.972
	PCR	1	1	0	69	1.0	0.986	0.972	0.972
B. fragilis	Culture	3	0	0	68	1.0	1.0	n/a	1.0
	PCR	3	0	0	68	1.0	1.0	1.0	1.0
C. freundii	Culture	0	0	1	70	0.0	1.0	n/a	0.986
	PCR	1	0	0	70	1.0	1.0	0.986	0.986
C. koseri	Culture	0	0	2	69	0.0	1.0	n/a	0.972
	PCR	2	0	0	69	1.0	1.0	0.972	0.972
C. novyi Group	Culture	0	0	0	71	n/a	1.0	n/a	0.986
	PCR	0	1	0	70	n/a	0.986	0.986	0.986
C. perfringens	Culture	0	0	1	70	0.0	1.0	n/a	0.986
	PCR	1	0	0	70	1.0	1.0	0.986	0.986
E. cloacae	Culture	0	0	4	67	0.0	1.0	n/a	0.915
	PCR	4	2	0	65	1.0	0.97	0.915	0.915
E. coli	Culture	4	0	2	65	0.667	1.0	1.0	0.955
	PCR	6	1	0	64	1.0	0.985	0.955	0.955
E. faecalis	Culture	0	0	26	45	0.0	1.0	0.0	1.0
	PCR	26	4	0	41	1.0	0.911	0.577	0.577
E. faecium	Culture	0	0	1	70	0.0	1.0	0.0	1.0
	PCR	1	2	0	68	1.0	0.971	0.958	0.958
K. oxytoca	Culture	0	0	2	69	0.0	1.0	0.0	1.0
	PCR	2	3	0	66	1.0	0.957	0.93	0.93
K. pneumoniae	Culture	1	0	5	65	0.167	1.0	1.0	0.929
	PCR	6	0	0	65	1.0	1.0	0.929	0.929
M. morganii	Culture	1	0	3	67	0.25	1.0	1.0	0.957
	PCR	4	0	0	67	1.0	1.0	0.957	0.957
P. aeruginosa	Culture	15	0	1	55	0.938	1.0	0.867	0.982
	PCR	14	0	2	55	0.875	1.0	0.867	0.982
P. mirabilis	Culture	0	0	3	68	0.0	1.0	n/a	0.93
	PCR	3	2	0	66	1.0	0.971	0.93	0.93
P. vulgaris	Culture	0	0	1	70	0.0	1.0	n/a	0.986
	PCR	1	0	0	70	1.0	1.0	0.986	0.986
S. agalactiae	Culture	5	0	1	65	0.833	1.0	1.0	0.924
	PCR	6	4	0	61	1.0	0.938	0.924	0.924
S. aureus	Culture	36	0	1	34	0.973	1.0	0.972	0.914
	PCR	36	2	1	32	0.973	0.941	0.972	0.914
S. lugdunensis	Culture	1	0	5	65	0.167	1.0	1.0	0.857
	PCR	6	5	0	60	1.0	0.923	0.857	0.857
S. maltophilia	Culture	0	0	2	69	0.0	1.0	n/a	0.944
	PCR	2	2	0	67	1.0	0.971	0.944	0.944
S. marcescens	Culture	1	0	1	69	0.5	1.0	1.0	0.986
	PCR	2	0	0	69	1.0	1.0	0.986	0.986

*PA_Sensitivity and PA_Specificity: Sensitivity and specificity from the probability-adjusted symmetric classification, where high-probability PCR-only detections (≥ 0.80) were reclassified as true positives. *CR_Sensitivity and CR_Specificity: PCR sensitivity and specificity when culture was used as the gold standard, with all PCR-only detections assumed to be false positives.

Supplemntary Table S13. Likelihood-adjusted performance reclassification metrics

Case_No	Strict Conventional model	Penalized PCR	Likelihood-Adjusted (PCR)	Likelihood-Adjusted (Culture)	ABR + Likelihood-Adjusted (PCR)	ABR + Likelihood-Adjusted (Culture)	ABR_Genes_Detected	High_Prob_Organisms (=>0.8 for GPC and => 0.5 for GNR)	Moderate_Prob_Organisms (GPC: 0.6-0.8/GNR 0.3-0.5)	Low_Prob_Organisms	Matched PCR and Culture organisms	Culture only organisms
1	Partial TP	FP	FP	TP	TP+R	FN+R	Erm(A)/Erm(B); Tet(M); Van Group; ampC	Escherichia coli; Enterobacter cloacae (Prob=0.636)	Enterococcus faecium (Prob=0.717)		Escherichia coli	
3	TP	TP	TP	TP	TP	TP	Erm(A)/Erm(B)	Staphylococcus aureus			Staphylococcus aureus	
4	TP	TP	TP	TP	TP	TP	mecA	Pseudomonas aeruginosa			Pseudomonas aeruginosa	
6	Partial TP	FN	FN	FN+	FN	FN+	Tet(M)	Streptococcus agalactiae			Streptococcus agalactiae	Staphylococcus aureus
7	FP	FP	TP+	FN+	TP+	FN+	Erm(A)/Erm(B); Tet(M); mecA	Enterococcus faecalis				
8	Partial TP	FP	FP	TP	FP	TP	Erm(A)/Erm(B); Mef(A); Tet(M); mecA			Enterococcus faecalis (Prob=0.285)	Candida parapsilosis	
10	TP	TP	TP	TP	TP	TP	Mef(A); Tet(M)			Staphylococcus aureus (Prob=0.506)	Staphylococcus aureus	
12	Partial TP	FP	FP+	FN+	FP+	FN+	Erm(A)/Erm(B); Tet(M); mecA	Enterococcus faecalis; Staphylococcus aureus	Streptococcus agalactiae (Prob=0.651)		Staphylococcus aureus	
13	FP	FP	FP	TN	TP+R	FN+R	Erm(A)/Erm(B); Tet(M)	Proteus mirabilis (Prob=0.643)	Enterococcus faecalis (Prob=0.651)			
15	Partial TP	FP	TP+	FN+	TP+	FN+	Erm(A)/Erm(B); Tet(M); mecA	Enterococcus faecalis; Staphylococcus aureus; Proteus mirabilis (Prob=0.769)			Staphylococcus aureus	
16	TP	TP	TP	TP	TP	TP	mecA	Staphylococcus aureus			Staphylococcus aureus	pantoea agglomerans (off-PCR panel)
17	TP	TP	TP	TP	TP	TP		Staphylococcus aureus			Staphylococcus aureus	
18	Partial TP	FP	TP+	FN+	TP+	FN+	Tet(M); mecA	Enterococcus faecalis; Pseudomonas aeruginosa			Pseudomonas aeruginosa	group g Streptococcus (off-PCR panel)
19	Partial TP	FP	TP+	FN+	TP+	FN+	Erm(A)/Erm(B); Mef(A); Tet(M); mecA	Enterococcus faecalis; Morganella morganii; Proteus mirabilis; Proteus vulgaris; Staphylococcus aureus; Streptococcus agalactiae			Staphylococcus aureus; Streptococcus agalactiae	
22	Partial TP	FP	TP+	FN+	TP+	FN+	Erm(A)/Erm(B); Tet(M); mecA	Enterococcus faecalis; Staphylococcus aureus			Staphylococcus aureus	

Supplemntary Table S13. Likelihood-adjusted performance reclassification metrics-continued

Case_No	Strict Conventional model	Penalized PCR	Likelihood-Adjusted (PCR)	Likelihood-Adjusted (Culture)	ABR + Likelihood-Adjusted (PCR)	ABR + Likelihood-Adjusted (Culture)	ABR_Genes_Detected	High_Prob_Organisms (=>0.8 for GPC and => 0.5 for GNR)	Moderate_Prob_Organisms (GPC: 0.6-0.8/GNR 0.3-0.5)	Low_Prob_Organisms	Matched PCR and Culture organisms	Culture only organisms
25	Unidentified Additional Organisms	TP_UAO	TP+	FN+	TP+	FN+	AAC(6)-Ib (aac(6)-Ib),Ant(3),APH(3)-VIb (aph(3)-VI; Erm(A)/Erm(B); Mef(A); Sul Group; Tet(M); ampC; mecA	Enterobacter cloacae; Enterococcus faecalis; Staphylococcus aureus; Streptococcus agalactiae; Viridans streptococci			Staphylococcus aureus; Streptococcus agalactiae	
27	TP	TP	TP	TP	TP	TP	Tet(M); mecA	Staphylococcus aureus			Staphylococcus aureus	
28	Partial TP	FP	TP+	FN+	TP+	FN+	Sul Group; Tet(M); mecA	Bacteroides fragilis; Enterococcus faecalis; Staphylococcus aureus			Staphylococcus aureus; Bacteroides fragilis	
29	FP	FP	TP+	FN+	TP+	FN+	Erm(A)/Erm(B); Mef(A); Sul Group; Tet(A)/Tet(B)/Tet(S); Tet(M); Van Group; mecA	Enterococcus faecalis; Staphylococcus aureus; citrobacter koseri; Klebsiella oxytoca (Prob=0.800)				
30	FP	FP	TP+	FN+	TP+	FN+	Tet(M); mecA	Staphylococcus lugdunensis				
33	FP	FP	TP+	FN+	TP+	FN+	mecA	Staphylococcus lugdunensis				
34	TP	TP	TP	TP	TP	TP	Erm(A)/Erm(B); Tet(M); mecA	Streptococcus agalactiae			Streptococcus agalactiae	
36	Partial TP	FP	TP+	FN+	TP+	FN+	Erm(A)/Erm(B); Mef(A); Tet(M); mecA	Pseudomonas aeruginosa; Staphylococcus aureus; Streptococcus agalactiae; Viridans streptococci	Staphylococcus lugdunensis (Prob=0.718)		Pseudomonas aeruginosa; Staphylococcus aureus	
39	TP	TP	TP	TP	TP	TP		Staphylococcus aureus			Staphylococcus aureus	
41	Partial TP	FP	TP+	FN+	TP+	FN+	Erm(A)/Erm(B); Mef(A); SHV; Tet(M); mecA	Enterococcus faecalis; Klebsiella pneumoniae; Staphylococcus aureus; Viridans streptococci			Staphylococcus aureus	
42	Partial TP	FN	FN	TP	FN	TP		Serratia marcescens			Serratia marcescens	Pseudomonas aeruginosa
44	FP	FP	TP+	FN+	TP+	FN+	Tet(M); mecA	Aerococcus urinae				
45	Unidentified Additional Organisms	TP_UAO	TP+	FN+	TP+	FN+	SHV; mecA	Klebsiella pneumoniae; Staphylococcus aureus			Staphylococcus aureus	
46	Unidentified Additional Organisms	TP_UAO	TP+	FN+	TP+	FN+	DHA; Erm(A)/Erm(B); Mef(A); Tet(M)	Bacteroides fragilis; Citrobacter koseri; Morganella morganii; Pseudomonas aeruginosa; Serratia marcescens			Pseudomonas aeruginosa; Bacteroides fragilis	

Supplemntary Table S13. Likelihood-adjusted performance reclassification metrics-continued

Case_No	Strict Conventional model	Penalized PCR	Likelihood-Adjusted (PCR)	Likelihood-Adjusted (Culture)	ABR + Likelihood-Adjusted (PCR)	ABR + Likelihood-Adjusted (Culture)	ABR_Genes_Detected	High_Prob_Organisms (=>0.8 for GPC and => 0.5 for GNR)	Moderate_Prob_Organisms (GPC: 0.6-0.8/GNR 0.3-0.5)	Low_Prob_Organisms	Matched PCR and Culture organisms	Culture only organisms
47	Partial TP	FP	TP+	FN+	TP+	FN+	Erm(A)/Erm(B); Tet(M); mecA	Enterococcus faecalis; Proteus mirabilis; Pseudomonas aeruginosa			Pseudomonas aeruginosa	
48	Partial TP	FP	TP+	FN+	TP+	FN+		Enterococcus faecalis; Staphylococcus aureus; Viridans streptococci; Stenotrophomonas maltophilia (Prob=0.677)			Staphylococcus aureus	
49	Partial TP	FP	TP+	FN+	TP+	FN+	mecA	Escherichia coli; Staphylococcus lugdunensis			Escherichia coli	
52	Unidentified Additional Organisms	TP_UAO	TP+	FN+	TP+	FN+	SHV; Tet(M); ampC	Enterobacter cloacae; Enterococcus faecalis; Klebsiella pneumoniae; Pseudomonas aeruginosa			Pseudomonas aeruginosa	
54	FP	FP	TP+	FN+	TP+	FN+	ampC; mecA	Enterobacter cloacae (Prob=0.723)				
55	Partial TP	FP	TP+	FN+	TP+	FN+	SHV; Sul Group; Tet(M); mecA	Clostridium perfringens; Enterococcus faecalis; Enterococcus faecium; Klebsiella pneumoniae; Staphylococcus aureus			Staphylococcus aureus	
56	Unidentified Additional Organisms	TP_UAO	TP+	FN+	TP+	FN+	Erm(A)/Erm(B); mecA	Staphylococcus aureus; Stenotrophomonas maltophilia (Prob=0.779)			Staphylococcus aureus	
57	Unidentified Additional Organisms	TP_UAO	TP+	FN+	TP+	FN+		Pseudomonas aeruginosa; Klebsiella oxytoca (Prob=0.694)			Pseudomonas aeruginosa	
58	Unidentified Additional Organisms	TP_UAO	TP+	FN+	TP+	FN+	CTX-M Group; DHA; Erm(A)/Erm(B); Sul Group; Tet(A)/Tet(B)/Tet(S); Tet(M); dfr Group; mecA	Enterococcus faecalis; Escherichia coli; Morganella morganii; Proteus mirabilis; Staphylococcus aureus			Staphylococcus aureus	
59	Partial TP	FP	FP+	FN+	TP+R	FN+R	Erm(A)/Erm(B); [illegible]	Bacteroides fragilis; [illegible]	Enterococcus faecium [illegible]		Bacteroides [illegible]	
61	FN	FN	FN	TP	FN	TP	Tet(M); mecA	Enterococcus faecalis				Pseudomonas aeruginosa
62	TP	TP	TP	TP	TP	TP	Tet(M); mecA	Pseudomonas aeruginosa			Pseudomonas aeruginosa	
67	Partial TP	FP	TP+	FN+	TP+	FN+	Sul Group; Tet(A)/Tet(B)/Tet(S); Tet(M); mecA	Enterococcus faecalis; Escherichia coli	Staphylococcus lugdunensis (Prob=0.765); Aerococcus urinae (Prob=0.651)		Escherichia coli	group g Streptococcus (off-PCR panel)
68	Partial TP	FP	FP	TP	FP	TP				C. novyi A/B_C. septicum_H. histolytica_P. sordellii group (Prob=0.110)	Candida glabrata	
69	Partial TP	FP	TP+	FN+	TP+	FN+	Erm(A)/Erm(B); Tet(M)	Enterococcus faecalis			Candida albicans	

Supplemntary Table S13. Likelihood-adjusted performance reclassification metrics-continued

Case_No	Strict Conventional model	Penalized PCR	Likelihood-Adjusted (PCR)	Likelihood-Adjusted (Culture)	ABR + Likelihood-Adjusted (PCR)	ABR + Likelihood-Adjusted (Culture)	ABR_Genes_Detected	High_Prob_Organisms (=>0.8 for GPC and => 0.5 for GNR)	Moderate_Prob_Organisms (GPC: 0.6-0.8/GNR 0.3-0.5)	Low_Prob_Organisms	Matched PCR and Culture organisms	Culture only organisms
70	Partial TP	FP	FP+	FN+	FP+	FN+	Tet(M)	Enterococcus faecalis; Escherichia coli	Staphylococcus aureus (Prob=0.685)		Escherichia coli	Bacteroides vulgatus (off-PCR panel)
71	Unidentified Additional Organisms	TP_UAO	TP+	FN+	TP+	FN+	AAC(6)-Ib (aac(6)-Ib),Ant(3),APH(3)-VIb (aph(3)-VI; Erm(A)/Erm(B); Mef(A); OXA Group; Sul Group; Tet(A)/Tet(B)/Tet(S); Tet(M); mecA	Enterococcus faecalis; Staphylococcus aureus; Stenotrophomonas maltophilia; acinetobacter baumannii	Streptococcus agalactiae (Prob=0.714)		Staphylococcus aureus	
73	TP	TP	TP	TP	TP	TP		Pseudomonas aeruginosa			Pseudomonas aeruginosa	
75	Unidentified Additional Organisms	TP_UAO	TP+	FN+	TP+	FN+	AAC(6)-Ib (aac(6)-Ib),Ant(3),APH(3)-VIb (aph(3)-VI; OXA Group; Sul Group; Tet(A)/Tet(B)/Tet(S); Tet(M)	Staphylococcus aureus; acinetobacter baumannii			Staphylococcus aureus	
78	Unidentified Additional Organisms	TP_UAO	TP+	FN+	TP+	FN+	Erm(A)/Erm(B); Tet(M); mecA	Enterococcus faecalis; Pseudomonas aeruginosa			Pseudomonas aeruginosa	
79	Partial TP	TP	TP	TP	TP	TP	Tet(M); mecA	Staphylococcus aureus		Staphylococcus lugdunensis (Prob=0.401)	Staphylococcus aureus	
81	TP	TP	TP	TP	TP	TP	DHA; Erm(A)/Erm(B); Tet(M); mecA	Morganella morganii; Peptostreptococcus anaerobius			Morganella morganii	
83	Partial TP	FP	TP+	FN+	TP+	FN+	Sul Group; Tet(M); mecA	Enterococcus faecalis; Staphylococcus aureus	Viridans streptococci (Prob=0.705)		Staphylococcus aureus	
84	Unidentified Additional Organisms	TP_UAO	TP	TP	TP	TP	mecA	Staphylococcus aureus			Staphylococcus aureus	
86	TP	TP	TP	TP	TP	TP	Tet(M)	Staphylococcus aureus			finegoldia magna; Staphylococcus aureus	
88	TP	TP	TP	TP	TP	TP	mecA			Staphylococcus aureus (Prob=0.580)	Staphylococcus aureus	
89	Partial TP	FP	TP+	FN+	TP+	FN+	ampC; mecA	Enterobacter cloacae; Pseudomonas aeruginosa; Staphylococcus aureus			Staphylococcus aureus	
92	Unidentified Additional Organisms	TP_UAO	FP	TP	TP+R	FN+R	Erm(A)/Erm(B); Tet(M); mecA	Escherichia coli (Prob=0.602)	Staphylococcus aureus (Prob=0.648); Streptococcus agalactiae (Prob=0.717)		Staphylococcus aureus	

Supplemntary Table S13. Likelihood-adjusted performance reclassification metrics-continued

Case_No	Strict Conventional model	Penalized PCR	Likelihood-Adjusted (PCR)	Likelihood-Adjusted (Culture)	ABR + Likelihood-Adjusted (PCR)	ABR + Likelihood-Adjusted (Culture)	ABR_Genes_Detected	High_Prob_Organisms (=>0.8 for GPC and => 0.5 for GNR)	Moderate_Prob_Organisms (GPC: 0.6-0.8/GNR 0.3-0.5)	Low_Prob_Organisms	Matched PCR and Culture organisms	Culture only organisms
93	Unidentified Additional Organisms	TP_UAO	TP+	FN+	TP+	FN+	BIL/LAT/CMY; Mef(A); Qnr Group; SHV; Sul Group; Tet(M)	Enterococcus faecalis; Escherichia coli; Klebsiella oxytoca; Klebsiella pneumoniae; Pseudomonas aeruginosa; Streptococcus agalactiae; citrobacter freundii	Staphylococcus aureus (Prob=0.759)		Pseudomonas aeruginosa; Staphylococcus aureus; Streptococcus agalactiae	Bacteroides fragilis group (off-PCR panel)
95	Unidentified Additional Organisms	TP_UAO	TP+	FN+	TP+	FN+	Erm(A)/Erm(B); Tet(M); ampC; mecA	Enterobacter cloacae; Enterococcus faecalis; Staphylococcus aureus			Staphylococcus aureus	
98	Unidentified Additional Organisms	TP_UAO	FP	FN+	TP+R	FN+R	Erm(A)/Erm(B); Tet(M); mecA	Enterococcus faecalis; Klebsiella oxytoca (Prob=0.676)	Staphylococcus aureus (Prob=0.689)		Staphylococcus aureus	
99	TP	TP	TP	TP	TP	TP				Staphylococcus lugdunensis (Prob=0.381)	Staphylococcus lugdunensis	
100	TP	TP	TP	TP	TP	TP				Staphylococcus aureus (Prob=0.178)	Staphylococcus aureus	
101	Partial TP	FP	TP+	FN+	TP+	FN+	Tet(M); mecA	Enterococcus faecalis; Pseudomonas aeruginosa; Staphylococcus lugdunensis			Pseudomonas aeruginosa	
103	TP	TP	TP	TP	TP	TP	Erm(A)/Erm(B); mecA	Staphylococcus aureus			Staphylococcus aureus	
104	Unidentified Additional Organisms	TP_UAO	TP+	FN+	TP+	FN+	SHV; Sul Group; Tet(M)	Klebsiella oxytoca; Pseudomonas aeruginosa; Stenotrophomonas maltophilia	Staphylococcus aureus (Prob=0.754); Streptococcus agalactiae (Prob=0.761); Enterococcus faecalis (Prob=0.742)		Pseudomonas aeruginosa; Staphylococcus aureus	
105	FP	FP	FP	TN	TP+R	FN+R	Tet(M); mecA		Enterococcus faecalis (Prob=0.626)	Staphylococcus lugdunensis (Prob=0.443)		
106	FP	FP	FP	TN	TP+R	FN+R	Tet(M); mecA		Staphylococcus aureus (Prob=0.622); Staphylococcus [illegible]			corynebacterium striatum (off PCR [illegible]
107	TP	TP	TP	TP	TP	TP	Erm(A)/Erm(B); mecA	Staphylococcus aureus			Staphylococcus aureus	
2	TN	TN	TN	TN	TN	TN						
5	TN	TN	TN	TN	TN	TN						
11	TN	TN	TN	TN	TN	TN						
14	TN	TN	TN	TN	TN	TN						
20	TN	TN	TN	TN	TN	TN						
21	TN	TN	TN	TN	TN	TN						
26	TN	TN	TN	TN	TN	TN						
35	TN	TN	TN	TN	TN	TN						

Supplemntary Table S13. Likelihood-adjusted performance reclassification metrics-continued

Case_No	Strict Conventional model	Penalized PCR	Likelihood-Adjusted (PCR)	Likelihood-Adjusted (Culture)	ABR + Likelihood-Adjusted (PCR)	ABR + Likelihood-Adjusted (Culture)	ABR_Genes_Detected	High_Prob_Organisms (=>0.8 for GPC and => 0.5 for GNR)	Moderate_Prob_Organisms (GPC: 0.6-0.8/GNR 0.3-0.5)	Low_Prob_Organisms	Matched PCR and Culture organisms	Culture only organisms
37	TN	TN	TN	TN	TN	TN						
43	TN	TN	TN	TN	TN	TN						
60	TN	TN	TN	TN	TN	TN						
63	TN	TN	TN	TN	TN	TN						
64	TN	TN	TN	TN	TN	TN						
65	TN	TN	TN	TN	TN	TN						
66	TN	TN	TN	TN	TN	TN						
74	TN	TN	TN	TN	TN	TN						
76	TN	TN	TN	TN	TN	TN						
80	TN	TN	TN	TN	TN	TN						
82	TN	TN	TN	TN	TN	TN						
87	TN	TN	TN	TN	TN	TN						
90	TN	TN	TN	TN	TN	TN						
91	TN	TN	TN	TN	TN	TN						
94	TN	TN	TN	TN	TN	TN						
96	TN	TN	TN	TN	TN	TN						
97	TN	TN	TN	TN	TN	TN						
